# Beyond Sensitivity: A Perspective on Transitioning Photonic Sensors into Real-Life Applications

**DOI:** 10.3390/s26196017

**Published:** 2026-09-23

**Authors:** Muhammad A. Butt

**Affiliations:** Institute of Microelectronics and Optoelectronics, Warsaw University of Technology, Koszykowa 75, 00-662 Warsaw, Poland; ali.butt@pw.edu.pl

**Keywords:** photonic sensors, real-world deployment, system integration, analytical validation, translational photonics

## Abstract

Photonic sensing encompasses technologies at markedly different levels of maturity. Fiber-optic sensing systems are already widely deployed in infrastructure, energy, transportation, and industrial monitoring, whereas many integrated biosensors and plasmonic, metasurface, and wearable platforms remain at laboratory or early translational stages. This review critically examines the factors that have enabled some photonic sensing technologies to achieve sustained deployment while continuing to constrain others. Practical performance is considered not only in terms of the optical transducer but also through molecular recognition, sample composition, environmental cross-sensitivity, fabrication variability, optical interrogation, microfluidics, packaging, calibration, electronics, and data processing. Established, transitional, and emerging platforms are comparatively assessed across healthcare, environmental monitoring, industrial process control, structural monitoring, and security applications. Real-life readiness is evaluated through analytical validity, selectivity, reproducibility, stability, dynamic range, response time, manufacturability, usability, and total system value. An application-driven framework is proposed in which sensing architecture, recognition chemistry, sample handling, interrogation, packaging, and validation are co-designed according to the intended operating environment. The analysis shows that successful deployment depends on robust system integration, reproducible manufacturing, application-specific validation, and a clear practical advantage rather than record sensitivity or nominal detection limits alone. Future evaluations should therefore use representative samples, independently manufactured devices, realistic operating conditions, standardized reporting protocols, and comparisons with established analytical methods.

## 1. Introduction

Photonic sensing has evolved from a specialized branch of optical measurement into a broad technological field spanning healthcare, environmental surveillance, food safety, industrial process control, structural health monitoring, and security [1]. By exploiting changes in refractive index, absorption, emission, scattering, phase, polarization, or resonance conditions, photonic sensors can convert molecular, chemical, biological, and physical perturbations into measurable optical signals [2,3]. Their inherent advantages, including high sensitivity, rapid response, label-free operation, immunity to electromagnetic interference, multiplexing capability, remote interrogation, and compatibility with miniaturized platforms, make them particularly attractive for applications in which conventional electrical or laboratory-based analytical methods are insufficient [4,5]. Advances in integrated photonics, optical fibers, resonant cavities, photonic crystals, plasmonic structures, metasurfaces, microfluidics, and functional materials have further expanded the range of detectable analytes and operating environments [6,7,8,9]. These technologies, however, do not occupy a common stage of development. Fiber Bragg grating, interferometric, and distributed fiber-optic sensors are already commercially established and deployed in infrastructure, energy, transportation, and industrial monitoring. Conversely, many integrated biosensors, metasurface devices, and wearable photonic platforms remain at laboratory or early translational stages. This review therefore does not assume a universal laboratory-to-field pathway; instead, it uses mature technologies as practical benchmarks for examining why other photonic sensing approaches have not achieved comparable deployment.

At the device level, considerable progress has been achieved. High-quality-factor resonators, slow-light waveguides, strongly confined electromagnetic modes, interferometric architectures, and surface-enhanced optical phenomena have enabled the detection of exceptionally small changes in refractive index, molecular concentration, strain, temperature, and other measurands [10,11,12]. In many studies, performance is primarily described through sensitivity, quality factor, resonance linewidth, figure of merit, or limit of detection [13,14,15]. These metrics are essential for characterizing the intrinsic response of a sensing element, but they provide only a partial assessment of its practical value. A sensor with record sensitivity under controlled laboratory conditions may still be unsuitable for real-world operation if its response is affected by temperature, fabrication variations, nonspecific adsorption, sample heterogeneity, optical coupling instability, environmental contamination, or signal drift [16]. Similarly, an extremely low limit of detection obtained using purified targets in optimized buffer solutions cannot be directly interpreted as evidence of clinical, environmental, or industrial readiness [13].

For emerging and transitional photonic platforms, this distinction exposes a persistent gap between device-level research and practical deployment. A real-life sensor is not merely a photonic structure that responds to an external perturbation. It is a complete analytical system incorporating target recognition, sample handling, optical excitation, signal acquisition, calibration, environmental compensation, data processing, packaging, and user interaction [1,17]. Each component can influence the effective detection limit and overall reliability. In biosensing, for example, the final response may be governed as strongly by receptor affinity, surface coverage, mass transport, biofouling, and matrix interference as by the optical sensitivity of the transducer [18]. Environmental sensors must operate in samples with variable pH, salinity, turbidity, and contaminant composition, while industrial and structural sensors must retain calibration under mechanical stress, temperature cycling, radiation, corrosion, or prolonged exposure [19]. Consequently, the transition from a photonic sensing element to a deployable product requires simultaneous optimization across optical, chemical, mechanical, electronic, and system-level domains [20].

The continued emphasis on maximum sensitivity can also obscure the importance of application-specific performance. Practical sensing rarely requires the highest physically achievable response. Instead, it requires sufficient sensitivity within a relevant concentration or measurement range, together with selectivity, accuracy, precision, rapid response, acceptable recovery, and long-term stability [21]. For many applications, a moderately sensitive sensor with a wide dynamic range, internal reference, simple readout, and reproducible fabrication may be more valuable than a highly sensitive device that requires precise wavelength control and complex laboratory instrumentation [22]. The appropriate performance hierarchy is therefore determined by the intended use. Clinical screening requires diagnostic sensitivity and specificity across representative patient populations [23]. Environmental monitoring prioritizes durability, low maintenance, and continuous operation. Industrial sensing demands reliability, traceability, and compatibility with established control infrastructure [24]. Evaluating these systems solely based on a single device-level metric is scientifically inadequate and may favor designs that demonstrate impressive laboratory performance but have limited translational potential.

This issue is becoming increasingly important as integrated, miniaturized, and multifunctional photonic platforms approach greater manufacturing maturity. Silicon, silicon nitride, indium phosphide, polymers, glass, and hybrid material systems offer complementary opportunities for scalable sensor fabrication [25,26,27,28]. Foundry-based manufacturing and process design kits are lowering barriers to photonic chip development, while advances in heterogeneous integration enable light sources, detectors, electronics, microfluidics, and functional materials to be incorporated into increasingly compact platforms. However, scalable fabrication alone does not ensure practical deployment. Device-to-device variability, packaging costs, fluidic integration, reproducible surface functionalization, storage stability, calibration protocols, and regulatory validation often remain less mature than the optical sensing element [29]. Progress toward real-world implementation therefore requires a shift from isolated component optimization to the concurrent engineering of the complete measurement chain.

This paper examines photonic sensing from the perspective of real-life operation rather than through a conventional catalogue of device architectures. Its principal contribution is an application-driven framework that connects device-level performance with analytical validation, system integration, field operation, scalable manufacturing, and commercial or regulatory requirements. Instead of asking only how optical sensitivity can be increased, the framework evaluates whether the achieved response is necessary, reproducible, stable, and usable under the conditions of intended operation. As summarized in Figure 1, successful translation requires coordinated consideration of the photonic transducer, recognition interface, sample handling, optical interrogation, packaging, calibration, data processing, manufacturability, and application-level value.

The remainder of the paper is organized as follows. Section 2 distinguishes the photonic sensing element from the complete measurement system and presents a staged pathway from device demonstration to practical deployment. Section 3 defines real-life performance through analytical validity, reliability, usability, and total system value. Section 4 compares resonant, interferometric, waveguide-engineered, fiber-optic, plasmonic, and metasurface platforms according to their translational strengths and limitations. Section 5 examines application-specific pathways in healthcare and point-of-care diagnostics, environmental and food-safety monitoring, and industrial and structural monitoring. Section 6 analyses the performance gap between controlled laboratory demonstrations and operation with real samples, variable environments, manufacturing variation, and prolonged use. Section 7 addresses the integration of optical, fluidic, chemical, electronic, mechanical, and computational subsystems as a central translational challenge. Section 8 considers scalable manufacturing, standardized evaluation, benchmarking, commercial viability, and regulatory requirements. Section 9 considers data-assisted analysis, standardized datasets and reporting, cartridge–reader architectures, and heterogeneous integration. Finally, Section 10 consolidates the principal conclusions and defines the evidence required for photonic sensors to progress from high-performance prototypes to reliable, manufacturable, and application-ready systems.

## 2. From Photonic Device to Real-World Sensing System

### 2.1. The Photonic Sensing Element

A photonic sensing element converts a physical, chemical, or biological interaction into a measurable modification of an optical field. The transduction mechanism is generally governed by changes in the complex refractive index of the sensing region. A variation in its real component modifies the effective refractive index, propagation constant, phase, polarization, or resonance condition, whereas a variation in its imaginary component influences optical absorption and propagation loss [30]. The resulting sensor response depends not only on the magnitude of the external perturbation but also on the spatial overlap between the optical mode and the region containing the target analyte.

Refractive index sensing can involve changes throughout the surrounding medium or changes confined to a molecular layer at the sensor surface [31]. In resonant devices, including microring resonators, microdisks, photonic crystal cavities, and coupled resonator structures, a change in effective refractive index modifies the optical path length and produces a measurable resonance wavelength shift [32]. The magnitude of this shift is determined by the field interaction with the analyte, material dispersion, and confinement of the optical mode [33]. Resonance linewidth and signal noise subsequently determine how accurately the shift can be resolved. In interferometric sensors, the analyte modifies the phase accumulated by light propagating through the sensing path [34]. Interference with an unperturbed reference path converts this phase difference into a measurable variation in output intensity. Mach Zehnder, Young, Michelson, and bimodal interferometers use this principle to detect refractive index, molecular binding, temperature, strain, and pressure [34]. Differential interrogation can also suppress common disturbances when the sensing and reference paths respond similarly to source fluctuations or environmental variations [35].

Absorption-based sensors detect wavelength-dependent optical attenuation associated with molecular, vibrational, or electronic transitions [36,37]. According to the Beer–Lambert relation, the transmitted intensity depends on analyte concentration, absorption coefficient, and interaction length [38]. Waveguides, resonant cavities, hollow core fibers, and slow-light structures can increase effective interaction between light and the absorbing medium. However, the resulting enhancement may be accompanied by increased optical loss, reduced bandwidth, or greater sensitivity to fabrication imperfections.

Fluorescence sensing relies on optical excitation of a fluorophore followed by emission at a longer wavelength [39,40]. Analyte information can be obtained from fluorescence intensity, emission spectrum, lifetime, or polarization. Photonic structures can enhance excitation, control emissions, and improve collection efficiency. Nevertheless, photobleaching, background fluorescence, labeling variability, and variations in fluorophore density can limit quantitative accuracy. Raman sensing instead detects inelastic scattering associated with molecular vibrational modes. The resulting spectrum provides chemically specific information without conventional fluorescent labels. Because spontaneous Raman scattering is weak, practical systems commonly employ long optical interaction lengths, resonant enhancement, or surface-enhanced Raman scattering [41].

These optical interactions must ultimately be converted into an observable signal. Wavelength interrogation measures displacement of a resonance or spectral feature, phase interrogation measures changes in optical path length, and intensity interrogation measures changes in transmitted, reflected, scattered, or emitted optical power [42]. Wavelength and phase measurements can provide high resolution but generally require stable sources, accurate spectral analysis, and controlled environmental conditions. Intensity measurements allow for simpler and more compact instrumentation but are more vulnerable to source power fluctuations, coupling variations, detector drift, and propagation loss [42]. Selection of the transduction and interrogation methods should therefore be guided by the required resolution, dynamic range, measurement speed, operating environment, and permitted system complexity.

### 2.2. Supporting System Components

The photonic sensing element constitutes only one component of the complete measurement chain. Practical performance depends on the compatibility and stability of the optical, fluidic, chemical, electronic, and computational subsystems surrounding it. Noise, drift, or variability introduced by these components may exceed the target-induced optical response and therefore determine the effective system-level detection limit. The optical subsystem must provide stable excitation, efficient coupling, and sufficiently precise signal acquisition. Source wavelength, linewidth, power, polarization, and temporal stability must be matched to the selected transduction and interrogation method [43]. Photodetector noise, responsivity, bandwidth, linearity, and dynamic range further constrain measurement precision [44]. Spectrally resolved measurements may require tunable lasers, detector arrays, or compact spectrometers, whereas fixed-wavelength or intensity-based interrogation can reduce instrumental complexity when compatible with the required resolution [42]. Optical interfaces and packaging must maintain reproducible coupling while limiting insertion loss, misalignment, polarization fluctuations, vibration, and environmental contamination.

For chemical and biological sensing, the sample-delivery and recognition interfaces determine whether the optical response remains analytically meaningful. Fluidic transport, mixing, filtration, enrichment, washing, and waste handling influence analyte recovery, binding kinetics, response time, and reproducibility. Surface functionalization provides molecular selectivity, but variations in receptor density, orientation, accessibility, biological activity, and nonspecific adsorption can introduce substantial uncertainty [45]. Temperature and other environmental variables must also be stabilized, referenced, or computationally compensated because they affect optical properties, detector response, and molecular interactions. Readout electronics control optical excitation and detection while providing signal amplification, filtering, digitization, timing, and communication. Subsequent processing may include baseline correction, spectral or phase extraction, resonance tracking, calibration, drift compensation, concentration estimation, and measurement-quality assessment [46,47]. These functions should be designed around a system-level error budget that identifies how uncertainty propagates across the complete measurement chain. The detailed integration requirements and interdependencies among these subsystems are discussed in Section 7.

### 2.3. A Proposed Technology Translation Pathway

The transition from a photonic sensing element to a practical product can be described through the seven-stage framework presented in Figure 2. Although these stages are shown in a defined sequence, the development process is inherently iterative. Limitations identified during real sample validation, field testing, or manufacturing frequently require modification of the original device, surface chemistry, optical interrogation method, or packaging approach. The framework is conceptual; proportions of technologies at each stage are not estimated because this review does not establish a systematic dataset with consistent stage-assignment criteria.

The first stage is demonstration of the photonic device under controlled conditions. At this level, the physical transduction principle is established using known refractive index changes or controlled optical, thermal, mechanical, chemical, or biological perturbations. Sensitivity, optical loss, resonance linewidth, dynamic range, noise, and repeatability are evaluated. This stage confirms that the device can detect the selected measurand, but it does not demonstrate application readiness [26]. The second stage is development of a functionalized sensing device. A selective recognition layer or responsive material is integrated with the photonic structure, and the response to the intended target is evaluated. Surface coverage, binding kinetics, selectivity, nonspecific adsorption, regeneration, and storage stability become important performance parameters. Appropriate negative controls and interfering species are required to distinguish specific target recognition from nonspecific optical changes [45].

The third stage involves integration of the sensing device with the necessary optical, fluidic, electronic, and computational components [48]. The assembled system should include the light source, detector, optical interface, sample delivery unit, readout electronics, thermal compensation, and data processing software. At this stage, performance must be assessed at the system level rather than inferred from measurements obtained using individual laboratory instruments. The fourth stage is validation using real samples. Clinical fluids, environmental samples, food matrices, industrial media, or representative structural conditions are introduced to determine whether the sensor retains its accuracy, selectivity, and dynamic range in complex operating environments [49,50]. Matrix effects, interferents, sample variability, recovery, precision, and false responses should be quantified. Results must also be compared with established reference methods to determine analytical agreement and practical benefit.

The fifth stage is field testing under conditions representative of the intended application. Temperature variation, humidity, vibration, contamination, transport, maintenance, calibration frequency, and user variability become central considerations. Field evaluation determines whether the performance observed during laboratory validation can be preserved during prolonged operation outside a controlled environment. The sixth stage addresses scalable production. Fabrication yield, dimensional tolerances, material uniformity, functionalization reproducibility, assembly, packaging, quality control, and unit cost must be evaluated across statistically meaningful production batches [51,52]. Designs optimized exclusively for maximum sensitivity may require modification if they are excessively vulnerable to normal manufacturing variations. The final stage is commercial or clinical deployment. The sensing system must demonstrate a clear advantage over existing analytical or measurement technologies in terms of accuracy, speed, portability, multiplexing, remote operation, safety, or total cost. Regulatory compliance, standardized manufacturing, supply chains, maintenance procedures, data management, and user training must also be established. Clinical deployment additionally requires evidence of diagnostic performance in representative patient populations and within the intended clinical workflow.

As illustrated in Figure 2, successful translation depends on preserving the analytical value of the optical response throughout functionalization, integration, validation, manufacturing, and routine operation. Sensitivity is therefore an essential starting parameter, but it is not an adequate measure of technological maturity. A deployable photonic sensor must transform the intrinsic response of a photonic element into reliable measurements and, ultimately, into information that supports a practical decision.

## 3. What Does “Real-Life Performance” Mean?

Real-life performance is the capacity of a complete sensing system to generate accurate, reproducible, and decision-relevant information under the conditions of its intended use. It is therefore a system-level property rather than an intrinsic characteristic of the photonic transducer. Although sensitivity, resonance linewidth, quality factor, and limit of detection (LOD) are essential descriptors of optical performance, they do not independently establish analytical validity or deployment readiness [13]. Practical performance additionally depends on molecular recognition, sample composition, transport kinetics, environmental cross-sensitivity, calibration stability, device-to-device variability, instrumentation, manufacturing yield, and user interaction. These factors should be evaluated collectively using predefined acceptance criteria derived from the target application. The principal distinctions between laboratory performance metrics and the corresponding validation requirements for real-life deployment are summarized in Table 1.

### 3.1. Analytical Performance in Representative Samples

Sensitivity describes the change in the measured output per unit change in analyte concentration or physical measurand. For resonant sensors, it is commonly expressed as a wavelength displacement per refractive-index unit or analyte concentration, whereas interferometric and intensity-based devices may report phase or normalized intensity changes [53]. Sensitivity alone does not define measurement resolution because the smallest detectable change also depends on signal noise, spectral resolution, linewidth, fitting uncertainty, baseline stability, and calibration variability. Consequently, a large optical response does not necessarily correspond to a low system-level LOD [54].

The LOD should represent the smallest analyte concentration or measurand that can be distinguished reliably from the blank response according to a specified statistical criterion. It should not be inferred solely from instrumental resolution or from the ratio between noise and the slope of an idealized calibration curve. A defensible LOD requires an experimentally determined blank distribution, sufficient independent replicates, a validated calibration model, and consideration of uncertainty near the lower end of the measurement range. The limit of quantification is equally important because it identifies the lowest concentration that can be measured with acceptable precision and bias. For many applications, reliable quantification around a clinically or operationally relevant decision threshold is more valuable than detection at an exceptionally low concentration.

Across photonic architectures, standardization should harmonize the validation procedure while retaining matrix-specific LOD and LOQ values. Wavelength, phase, and intensity responses should be converted into analyte concentration through matrix-matched calibration covering the complete analytical workflow. A common protocol should specify false-positive and false-negative probabilities for LOD determination using blanks and low-concentration samples, and predefined precision and bias limits for experimental verification of LOQ [55]. Validation should include independent sample preparations, devices, and measurement days, with acquisition bandwidth, averaging time, drift correction, and replicate numbers reported. Where analyte-free matrices are unavailable, surrogate or analyte-depleted samples require demonstrated suitability. Direct comparisons should therefore use the same analyte, representative matrix, and statistical criteria, while distinguishing instrumental refractive-index resolution from assay-level concentration limits.

Performance measured in purified targets or controlled buffer solutions may deteriorate substantially in serum, plasma, whole blood, saliva, urine, environmental water, food extracts, or industrial media [56]. These matrices contain species that can modify bulk refractive index, absorb or scatter the interrogating light, occupy receptor sites, foul the surface, or alter target transport and binding kinetics. Variations in pH, ionic strength, viscosity, turbidity, and sample composition can further change both the optical baseline and the activity of the recognition layer. Matrix-matched calibration, spike-and-recovery analysis, dilution linearity, and comparison with an established reference method are therefore required to establish whether the analytical response remains valid in representative samples.

Physical sensitivity must also be distinguished from chemical or biological selectivity. A photonic structure may respond strongly to any perturbation that modifies the local refractive index or optical loss, but it cannot inherently determine the molecular origin of that change [7]. Target selectivity is introduced primarily by the recognition interface, including antibodies, aptamers, nucleic-acid probes, enzymes, molecularly imprinted polymers, or target-responsive materials. Its performance depends on receptor affinity, surface density, orientation, accessibility, binding kinetics, and stability. High receptor loading does not necessarily improve analytical performance because steric hindrance, transport limitation, and increased nonspecific adsorption may reduce the fraction of accessible binding sites.

Selectivity should be quantified using structurally related species, abundant matrix components, and other interferents expected in the intended application. Interferents should be tested individually and, where appropriate, in mixtures at realistic concentrations. Negative controls, reference surfaces, nonbinding receptors, blocking layers, and differential measurement channels are necessary to separate specific recognition from bulk-medium changes and nonspecific surface accumulation [57]. Cross-sensitivity to temperature, pH, salinity, humidity, pressure, and mechanical strain should likewise be characterized because these variables may produce optical responses comparable to or greater than the target-induced signal.

The dynamic range must encompass the concentration or measurand interval relevant to the intended decision [58]. A sensor with an exceptionally low LOD may be unsuitable if its response saturates below the clinically or industrially important range. Conversely, a broad dynamic range is of limited value if resolution is insufficient near the decision threshold. Calibration models should therefore be validated across the complete working range, including nonlinear regions, and their residual errors and uncertainty should be reported. The importance of evaluating sensor performance within clinically relevant decision intervals is illustrated by the multiplexed microring measurements shown in Figure 3 [59]. Across 37 single-donor plasma samples, the anti-VWF channel (Figure 3a) and anti-FVIII channel (Figure 3b) differentiated low-, normal-, and high-VWF groups. In contrast, the GP1b channel (Figure 3c), rat-tail collagen type I channel (Figure 3d), and human collagen type I channel (Figure 3e) more consistently distinguished low-VWF samples from the other groups but showed weaker or nonsignificant separation between normal and high concentrations [59]. These channel-dependent responses demonstrate that analytical performance cannot be represented by a single sensitivity or LOD value. Instead, it must be evaluated using representative samples, appropriate recognition chemistries, replicate distributions, validated working ranges, and predefined decision thresholds [59].

Response time should be defined at the system level rather than by the intrinsic speed of the optical field. The relevant interval includes sample introduction, analyte transport, molecular recognition, equilibration, washing, optical acquisition, signal processing, and result generation [60]. For flow-through sensors, the measured response may be limited by convection, diffusion, boundary-layer thickness, and association kinetics rather than by optical interrogation [61]. The required response time is application-dependent: emergency diagnostics and process control may require results within minutes or seconds, whereas long-term environmental and structural monitoring may prioritize stability and measurement continuity.

### 3.2. Reliability Across Devices, Time, and Operating Conditions

Accuracy expresses the closeness of a measurement to an accepted reference value, while precision describes the dispersion among repeated measurements. These parameters should be assessed independently because high precision can coexist with substantial systematic bias. Accuracy should be evaluated using certified reference materials, recovery experiments, or comparison with an established analytical method. Method-comparison studies should examine both correlation and agreement since a high correlation coefficient alone does not exclude proportional or constant bias [62].

Repeatability refers to variation under nominally identical conditions, such as repeated measurements using the same device, operator, and instrument over a short period. Intermediate precision extends the evaluation across days, operators, instruments, and environmental conditions within one laboratory [63]. Reproducibility further examines agreement among laboratories, production sites, or independently manufactured device batches. Demonstrating only repeated measurements on a single optimized sensor can underestimate variability arising from waveguide dimensions, coupling gaps, layer thickness, surface roughness, material properties, functionalization density, microfluidic assembly, and optical packaging.

Performance should therefore be reported for statistically meaningful numbers of independently fabricated and functionalized devices. Mean values alone are inadequate; standard deviation, coefficient of variation, confidence intervals, device yield, failure rate, and exclusion criteria should also be provided. Hierarchical analysis can help distinguish measurement-level, device-level, batch-level, and operator-level contributions to total variability [64]. Such information is essential for defining manufacturing tolerances and determining whether calibration can be transferred among devices or must be performed individually. Stability includes short-term baseline stability, resistance to environmental perturbations, long-term operational stability, and storage stability. Source-power fluctuations, polarization changes, detector noise, wavelength-reference drift, coupling instability, and electronic offsets may affect the readout even when the sensing element remains unchanged. Resonant and interferometric devices are particularly sensitive to temperature because thermo-optic and thermal-expansion effects modify the effective optical path length. Variations in the sample refractive index with temperature can introduce an additional matrix-dependent contribution.

Functionalized sensors may undergo receptor denaturation, hydrolysis, oxidation, desorption, surface reorganization, and progressive biofouling [65,66]. Repeated regeneration can also reduce receptor activity or alter the underlying surface. Stability studies should therefore examine changes in baseline, sensitivity, selectivity, calibration slope, noise, and recovery over the intended storage and operating periods. Shelf-life claims should be supported by measurements performed under defined storage conditions using independently prepared devices. Drift should be quantified as a time-dependent change in the blank or reference response and distinguished from random noise. Internal references, differential channels, integrated temperature sensors, wavelength standards, ratiometric measurements, and common-mode rejection can reduce systematic disturbances [67]. Reference channels must nevertheless be designed carefully because they should reproduce environmental and nonspecific effects without responding to the target. An inadequately matched reference may be introduced rather than remove measurement bias.

Calibration establishes the relationship between sensor output and the measurand. Its validity depends on the calibration material, matrix, environmental conditions, instrument configuration, device batch, and selected mathematical model [68,69]. Calibration transfer among devices requires evidence that unit-to-unit variation remains within an acceptable range. Otherwise, individual calibration may increase cost and operational complexity. Automated baseline correction or machine-learning models may compensate for predictable nonlinearities and multidimensional interference, but they should be trained and validated using independent, representative datasets. Computational correction cannot substitute for stable instrumentation, controlled surface chemistry, or traceable reference measurements.

### 3.3. Practicality, Usability, and Total System Value

A deployable sensor must be compatible with the complete workflow in which the measurement is performed. Relevant requirements include sample volume, sample preparation, measurement time, portability, energy consumption, maintenance, calibration frequency, waste management, operator competence, and biosafety [70,71]. The output should be converted into an interpretable quantity, threshold decision, alarm, or classification accompanied by appropriate quality indicators. Raw spectral changes are rarely sufficient for non-specialist users. Usability should therefore be evaluated through end-to-end operation by representative users rather than inferred solely from experiments conducted by device developers.

Packaging frequently dominates the complexity and cost of a practical photonic sensor. Stable optical coupling, fluidic sealing, thermal management, electrical connections, mechanical protection, and exposure of the sensing region must be achieved simultaneously [72]. An experimental example was presented by Adamopoulos et al., who integrated multilayer microfluidics with a monolithic electronic–photonic biosensor [29]. As illustrated in Figure 4a, selective backside removal of the silicon substrate exposed the photonic sensing region to the fluidic sample. The complete architecture in Figure 4b simultaneously accommodated fiber-optic access, fluid phot delivery, flip-chip electrical coupling, and glass-supported mechanical stabilization [29]. The use of vertically interconnected microfluidic layers also enabled densely spaced microscale channels at the sensor surface to interface with larger external tubing, thereby addressing the dimensional mismatch between millimeter-scale photonic chips and macroscopic sample-handling components [29].

Practical value must be assessed across the complete measurement workflow rather than from the photonic chip alone. The analysis should include optical instrumentation, sample preparation, reagents, disposable components, calibration, quality control, operator time, maintenance, data management, waste disposal, and the cost of invalid or repeated measurements. These contributions should be considered together with measurement accuracy, time to result, portability, operating lifetime, and the consequences of incorrect or delayed decisions. A low-cost sensing element does not necessarily produce an economical system if it depends on expensive interrogation equipment, frequent recalibration, specialist operation, or labor-intensive sample processing [73]. Ultimately, real-life performance is application-specific. It is achieved when the complete sensing system provides sufficient analytical capability within the relevant range, maintains traceable performance across devices and operating conditions, fits the intended workflow, and offers a measurable advantage over the established method. A balanced and reproducible system is therefore more valuable than an isolated device exhibiting record sensitivity under idealized conditions.

**Table 1:** Multidimensional framework for evaluating laboratory performance and real-life deployment readiness of photonic sensing systems.

Performance Dimension	Common Laboratory Metric or Practice	Principal Limitation of Laboratory Evaluation	Real-Life Performance Requirement
Sensitivity [15,31,74]	Spectral shift, phase change, or intensity variation per unit measurand	Does not account for noise, drift, matrix effects, or calibration uncertainty	Sufficient response within the application-specific range and around relevant decision thresholds
Detection capability [13,14]	LOD estimated from instrumental resolution or baseline noise in buffer	May underestimate blank variability and systematic error	Experimentally validated LOD and limit of quantification in representative samples
Selectivity and specificity [75,76]	Response to a purified target after receptor immobilization	Does not establish discrimination against nonspecific binding or interferents	Target-specific response in the presence of realistic matrix components and cross-reactive species
Accuracy [77,78,79]	Agreement with prepared standard concentrations	Standards may not reproduce analyte recovery or matrix-dependent bias	Traceable agreement with accepted values or reference methods
Precision [80,81,82]	Repeated measurements using one device	Captures only short-term measurement variation	Acceptable repeatability, intermediate precision, and reproducibility
Dynamic range [83,84]	Broad calibration range under controlled conditions	May not provide adequate resolution near decision thresholds or may include nonphysical extrapolation	Validated working range covering expected sample concentrations or operating conditions
Response time [85,86]	Optical acquisition time or initial signal rise	Excludes transport, binding, washing, preparation, and analysis	End-to-end time from sample introduction to an actionable result
Stability and drift [87,88,89]	Short measurement under constant conditions	Does not represent prolonged use, storage, transport, or environmental variation	Stable calibration and baseline over the intended operational and storage periods
Environmental robustness [87,90]	Operation at controlled temperature and humidity	Conceals cross-sensitivity to environmental variables	Accurate operation across the specified environmental envelope
Calibration [91,92]	Device-specific calibration immediately before measurement	May be impractical in routine use and may not transfer across devices	Traceable, stable, and operationally feasible calibration strategy
Optical interrogation [3,42,93]	Tunable laser, spectrum analyzer, polarization controller, and manual alignment	High cost, size, complexity, and operator dependence	Robust interrogation compatible with the required portability, resolution, and automation
Sample handling [94]	Manually prepared, filtered, or diluted sample	Preparation may dominate time, error, and analyte loss	Standardized or automated treatment of representative sample volumes
Manufacturability [95,96,97]	Successful fabrication of a small number of devices	Does not reveal tolerance sensitivity or production losses	Scalable production with acceptable yield and unit-to-unit variation
Packaging and integration [29,72,98]	Open chip with laboratory coupling and external fluidics	Omits alignment, sealing, thermal management, and protection requirements	Stable optical, fluidic, electrical, and mechanical integration
Cost and sustainability [95,99,100]	Cost of the photonic chip or sensing material	Excludes instrumentation, assembly, reagents, labor, maintenance, and disposal	Competitive total cost per valid result over the system lifetime

## 4. Photonic Platforms with Translational Potential

Photonic sensing platforms can be classified by their transduction architecture, light–matter interaction, or implementation format, although these categories often overlap. Platform selection should therefore prioritize application-specific analytical performance, reproducibility, stability, affordability, and deployment robustness rather than intrinsic sensitivity alone. Table 2 summarizes these distinctions, while Figure 5 compares the operating principles, advantages, and translational limitations of the major platforms.

### 4.1. Integrated Resonant Sensors

Integrated resonant sensors include microring and microdisk resonators, photonic-crystal cavities, resonant Sagnac-loop structures, and coupled-cavity configurations [15,101,102,103]. Their operation relies on the perturbation of an optical resonance by a change in effective refractive index, absorption, temperature, strain, or surface loading. Repeated optical circulation or strong field localization increases the effective interaction with the measurand, allowing for compact sensing regions and dense integration of multiple functionalized channels. Resonant arrays are therefore attractive for multiplexed biochemical assays, multivariable physical sensing, and systems requiring a small chip footprint. The practical performance of a resonator is not determined by quality factor or bulk sensitivity alone. The detectable resonance displacement also depends on optical power, resonance contrast, linewidth, spectral noise, fitting uncertainty, thermal drift, and the stability of the coupling condition. High-Q cavities may improve spectral resolution but are also more susceptible to fabrication disorder, surface contamination, temperature fluctuations, and resonance detuning [12]. Variations in waveguide width, cavity dimensions, material index, sidewall roughness, and coupling gap can modify the resonance wavelength, extinction ratio, and loaded quality factor across a wafer or fabrication batch [11]. Practical implementation therefore requires robust coupling conditions, reference resonators, temperature compensation, automated resonance tracking, and calibration procedures that account for device-to-device variability.

For high-volume manufacturing, differential readout using a nearby reference resonator is particularly attractive because it requires no additional material platform and can share the sensing resonator’s fabrication and interrogation infrastructure. Compensation should use separately calibrated thermal sensitivities, since differences in cladding or functionalization can prevent complete drift cancellation. A fully clad reference primarily tracks chip temperature, whereas a fluid-exposed reference with a nonbinding surface treatment can also capture temperature-dependent sample effects. Where supported by the foundry process, local diode-based thermometry provides complementary temperature measurements for numerical correction or integrated-heater feedback [104]. Active stabilization adds power consumption and thermal crosstalk, while compensating athermal coatings introduce additional processing and reliability requirements. Calibrated differential referencing therefore offers a practical starting point for limiting manufacturing complexity, with electrical thermometry or heating added according to the required measurement uncertainty.

### 4.2. Interferometric Sensors

Mach–Zehnder, Young, Michelson, bimodal, and conventional Sagnac interferometers convert measurand-induced changes in effective refractive index or optical path length into a differential phase response [105]. Unlike resonant devices, their sensitivity can be increased by extending the interaction length without necessarily relying on an ultranarrow spectral feature. The availability of a reference path, reference mode, or counterpropagating wave also facilitates common-mode rejection and differential compensation of source fluctuations, bulk-medium variations, and environmental disturbances. This architectural flexibility is advantageous for label-free biochemical sensing and for measurements of temperature, pressure, strain, displacement, acoustic fields, and rotation.

However, increasing the optical path length also enlarges the footprint and can increase propagation loss, phase noise, and sensitivity to structural nonuniformity. Interferometric outputs may be affected by polarization fluctuations, imperfect power splitting, path imbalance, and temperature-induced phase drift [4]. At large accumulated phase shifts, the periodic transfer function can additionally introduce phase ambiguity. Reliable implementation consequently requires stable splitting and recombination, effective referencing, phase-unwrapping or quadrature-readout methods, and packaging that preserves optical-path stability. For long-term drift control, hardware-based common-mode rejection should provide the primary safeguard, with software correcting residual differential drift. This approach requires matching the environmental responses of the sensing and reference paths, rather than their geometry alone [46]. Software correction should use reference signals and independently measured environmental variables, since processing the sensing signal alone may inadvertently remove slowly varying analyte responses. Data-assisted models should therefore be validated across independent devices and extended operating periods before being relied upon for long-term compensation.

### 4.3. Waveguide-Engineered Sensors

Waveguide engineering enhances sensing by increasing the fraction of the optical mode exposed to the analyte, modifying group velocity, or allowing the sample to penetrate the guiding structure [106]. Slot waveguides concentrate the electric field within a low-index nanoscale gap, whereas subwavelength-grating waveguides use deeply subwavelength periodicity to tailor modal confinement, anisotropy, and evanescent-field penetration [107]. Slow-light structures increase the interaction per unit propagation length through dispersion engineering, while porous waveguides provide a large internal surface and volumetric interaction with infiltrating species. These approaches can substantially strengthen light–matter interaction without requiring a proportionate increase in device footprint. The practical realization of these concepts is illustrated in Figure 6a–e, which present SEM images of fabricated SiN resonant-cavity sensors employing ridge, slot, photonic-crystal, and subwavelength-grating waveguide geometries [108]. The images show the substantial differences in structural complexity among these geometries, including the narrow low-index gap of the slot waveguide and the precisely patterned periodic features of the photonic-crystal and subwavelength-grating structures. Their successful fabrication demonstrates that multiple mode-engineering strategies can be implemented on a common material platform, while also emphasizing the lithographic fidelity and dimensional control required to preserve the intended modal confinement and resonant response [108].

Nevertheless, the optical enhancement is often accompanied by tighter fabrication tolerances, increased scattering loss, and more demanding surface processing. Narrow slots and nanoscale voids may be difficult to functionalize uniformly and can trap bubbles, particulates, or biomolecular aggregates. Slow-light structures are particularly sensitive to disorder-induced backscattering and may provide enhancement only over a restricted spectral bandwidth [109]. Porous structures can exhibit incomplete infiltration, slow diffusion, hysteresis, and difficult regeneration. Their translational feasibility must therefore be assessed using fabricated and functionalized devices under realistic flow conditions rather than inferred solely from simulated field overlap or group-index enhancement [108].

### 4.4. Fiber-Optic Sensors

Fiber-optic platforms include fiber Bragg gratings, Fabry–Pérot and other interferometric fiber sensors, distributed scattering-based systems, and specialty fibers such as microstructured, multicore, tapered, exposed-core, and photonic-crystal fibers [110,111,112,113]. Depending on the architecture, the measurand is encoded as a change in wavelength, phase, intensity, polarization, propagation loss, or backscattered signal. Multiple fiber sensors can be wavelength-, time-, frequency-, or spatially multiplexed, while distributed Rayleigh-, Raman-, and Brillouin-based methods can recover temperature, strain, or vibration continuously along an extended fiber. Their principal translational advantage is the ability to perform electrically passive measurements over long distances and in locations affected by strong electromagnetic fields, high voltage, limited access, or explosion risk. They are particularly suitable for structural-health monitoring, energy infrastructure, transportation systems, geotechnical installations, and industrial process monitoring. However, reliable deployment depends on more than the optical fiber itself [114]. Mechanical attachment, strain transfer, bending, connector losses, coating degradation, moisture ingress, and bonding-layer creep can all influence the measured response. Cross-sensitivity between temperature, strain, pressure, and surrounding refractive index must also be resolved through sensor design or compensation [115,116]. Long-term field validation and installation-specific calibration are therefore central to fiber-sensor translation.

### 4.5. Plasmonic and Metasurface Sensors

Plasmonic sensors exploit collective free-electron oscillations at metal–dielectric interfaces to generate strongly confined electromagnetic fields. Metasurface sensors use arrays of subwavelength metallic, dielectric, or hybrid resonators to engineer spectral, phase, polarization, or spatial responses [7,117,118]. Although their underlying resonance mechanisms differ, both platforms can provide strong surface sensitivity, compact planar geometries, and high-density arrays for parallel detection. Their compatibility with spectral, angular, polarization-resolved, or imaging-based interrogation creates opportunities for multiplexed surface assays and portable sensing systems using compact light sources and cameras. Plasmonic confinement is accompanied by absorption loss, resonance broadening, local heating, and possible degradation or contamination of the metallic surface [6]. Dielectric metasurfaces can reduce absorptive loss and support narrow Fano, guided-mode, Mie-type, or quasi-bound-state-in-the-continuum resonances, but high-Q responses may become highly sensitive to disorder, illumination angle, polarization, and environmental drift. In both cases, nanoscale variations in feature dimensions can produce substantial spectral nonuniformity across an array [119]. Surface stability, receptor immobilization, scalable nanofabrication, and reproducible large-area optical response are therefore as important as nominal refractive-index sensitivity. These platforms are especially promising for disposable sensor surfaces combined with reusable imaging or spectral readers.

### 4.6. Selecting a Platform According to the Application

No single photonic platform simultaneously maximizes sensitivity, selectivity, dynamic range, stability, multiplexing density, spatial coverage, manufacturability, and affordability. Platform selection should begin with the measurand and its expected range, the physical and chemical properties of the sample, the required response time, environmental variability, measurement location, service life, and acceptable instrumentation cost [120,121]. The sensing architecture should then be selected together with its recognition layer, sample-delivery method, reference strategy, readout hardware, packaging, and calibration procedure. Integrated resonators are attractive when compactness and dense multiplexing are priorities, whereas interferometers provide flexible interaction lengths and differential phase measurements. Waveguide-engineered structures are valuable when enhanced optical overlap is required, provided that their fabrication and fluidic accessibility remain manageable [122]. Fiber-optic systems are preferred for remote, distributed, or electromagnetically harsh environments, while plasmonic and metasurface devices offer compelling routes toward planar arrays and imaging-based surface assays [123]. A moderately sensitive platform compatible with stable, low-cost interrogation may ultimately provide greater translational value than a record-performing device that requires active alignment, high-resolution spectroscopy, stringent temperature control, or individual calibration. The optimal platform is therefore the one that satisfies predefined application-level requirements with the lowest overall technical and operational burden.

The translational status of photonic sensing is determined less by the optical principle alone than by the maturity of the complete measurement architecture. Fiber Bragg grating and distributed fiber-optic sensors are clear examples of successful deployment. Their remote, passive, electromagnetically immune, long-distance, and multiplexed operation suits infrastructure, energy, transportation, and industrial monitoring [114,124]. Their translation has been enabled by standardized fibers and interrogators, established installation practices, durable packaging, well-defined calibration procedures, and a clear economic benefit from continuous condition monitoring. In these applications, the value of the measurement is retained even when the sensor response is moderate, because the technology provides information that is difficult or costly to obtain by conventional electrical methods.

In contrast, integrated photonic biosensors, plasmonic sensors, metasurface sensors, and many waveguide-engineered platforms remain predominantly transitional technologies [6,125,126]. Their compact footprint, multiplexing potential, and strong light–matter interaction are scientifically compelling, but their practical performance is often limited by factors external to the photonic transducer: variable surface functionalization, biofouling, complex sample preparation, temperature-dependent baselines, optical packaging, and the requirement for reliable low-cost interrogation. Translation is therefore most likely when these platforms are developed for a narrowly defined analytical decision and are supported by a complete assay, cartridge, reference strategy, and reader architecture. The relevant comparison is not between optical platforms in isolation, but between complete sensing systems and the established method used for the same decision.

**Table 2:** Application-oriented comparison of major photonic sensing platforms and their translational requirements.

Platform Class	Representative Architectures	Dominant Optical Response	Main Translational Strengths	Principal Limitations and Cross-Sensitivities	Instrumentation and System Requirements	Best-Aligned Applications
Integrated resonant sensors [11,25,127]	Microrings, microdisks, photonic-crystal cavities, resonant Sagnac loops, coupled cavities	Resonance wavelength, intensity, phase, linewidth or mode splitting	Compact footprint; strong effective interaction; dense multiplexing; compatibility with integrated photonics	Fabrication-induced detuning; thermal drift; coupling variation; surface-loss sensitivity; restricted spectral range	Spectrally resolved or wavelength-locked interrogation; polarization and temperature control; reference channels	Multiplexed biosensing, compact chemical analysis and chip-scale physical sensing
Interferometric sensors [34,35,82,128]	Mach–Zehnder, Young, Michelson, bimodal and Sagnac interferometers	Differential phase or interference intensity	Adjustable interaction length; differential measurement; broad architectural flexibility; no requirement for ultrahigh-Q resonance	Large footprint; phase ambiguity; polarization sensitivity; path imbalance; thermal and mechanical drift	Stable splitting and recombination; reference arm or mode; phase recovery, quadrature detection or phase unwrapping	Label-free biochemical analysis and precision measurement of strain, pressure, acoustic fields, displacement, or rotation
Waveguide-engineered sensors [108,109]	Slot, subwavelength-grating, slow-light and porous waveguides	Effective-index, phase or attenuation change enhanced by modal overlap or dispersion	Strong field–analyte overlap; compact interaction region; engineerable dispersion and surface accessibility	Tight fabrication tolerances; scattering and backreflection; limited bandwidth; difficult functionalization; infiltration and fouling effects	Resonant, interferometric or intensity-based readout; controlled sample delivery; optimized surface processing	Small-volume biochemical or chemical assays requiring enhanced interaction
Fiber-optic sensors [124,129,130]	Fiber Bragg gratings, Fabry–Pérot and other fiber interferometers, distributed sensors, specialty fibers	Wavelength, phase, intensity, polarization, or backscattering variation	Remote and passive operation; electromagnetic immunity; long-distance multiplexing; distributed coverage; mechanical flexibility	Temperature–strain cross-sensitivity; bending; attachment-dependent response; coating and connector degradation	Remote optical interrogator, reflectometer, or coherent receiver; installation-specific calibration and compensation	Structural, industrial, energy, transportation, geotechnical and harsh-environment monitoring
Plasmonic and metasurface sensors [125,131,132,133,134]	Propagating and localized plasmonic structures; metallic, dielectric and hybrid metasurfaces	Spectral, angular, phase, polarization, intensity or image variation	Strong surface confinement; planar format; high-density arrays; imaging-compatible and potentially compact readout	Absorption loss and local heating in plasmonic structures; sensitivity to dimensional nonuniformity, illumination angle, polarization, surface contamination, and environmental drift	Spectral, angular, polarization-resolved, or imaging-based interrogation; controlled illumination; reference regions; stable functionalization and antifouling treatment	Multiplexed surface-binding assays, biomarker detection, compact imaging-based analysis, food and environmental screening, and disposable sensing surfaces with reusable readers

## 5. Translation Pathways for Real-Life Applications

Translation pathways should be defined according to both application requirements and technological maturity. Mature fiber-optic technologies are generally optimized through installation, interrogation, reliability qualification, and service-life validation, whereas emerging integrated, plasmonic, metasurface, and waveguide-engineered sensors require simultaneous development of the transducer, recognition chemistry, sample handling, reference strategy, packaging, and readout. The following examples therefore illustrate not a universal pathway for all photonic sensors, but the distinct evidence needed for mature and emerging technologies to provide useful real-life measurements.

### 5.1. Healthcare and Point-of-Care Diagnostics

Photonic biosensors can detect clinically relevant proteins, nucleic acids, extracellular vesicles, pathogens, circulating cells, metabolites, and inflammatory biomarkers [126,131,135,136]. Their potential for label-free, multiplexed, and real-time analysis makes them attractive for early diagnosis, therapeutic monitoring, and decentralized testing. Analytical detection alone, however, does not establish clinical utility. The measured biomarker must inform diagnosis or treatment, and the complete sensing system must provide adequate selectivity, accuracy, reproducibility, and dynamic range at clinically relevant concentrations [17].

Clinical specimens are considerably more complex than purified laboratory solutions. Serum and plasma contain abundant proteins, lipids, salts, and endogenous interferents that may cause biofouling, nonspecific adsorption, and matrix-dependent optical shifts. Whole blood introduces additional complications associated with cellular components, coagulation, absorption, and scattering, whereas saliva and urine vary with collection method, hydration, diet, and physiological state [49,137,138]. These factors can modify receptor activity and binding kinetics or generate responses unrelated to the target concentration. Reliable analysis may therefore require filtration, separation, dilution, enrichment, washing, antifouling coatings, or internal reference channels. Such operations must be integrated without imposing laboratory-level complexity on the end user [139].

A practical point-of-care system should combine the photonic transducer with controlled sample handling, stable optical interrogation, compact instrumentation, and automated data processing [126]. Multiplexing can improve diagnostic confidence by measuring biomarker panels together with positive, negative, and reference channels. However, it also requires uniform surface functionalization, reproducible fluid delivery, effective cross-reactivity control, and sensor-specific calibration [140,141]. The optical response must ultimately be converted into clinically interpretable information, such as a biomarker concentration, diagnostic classification, or risk score, rather than reported only as a wavelength or intensity shift. A complementary perspective is provided by Chen et al., who discuss clinical translation pathways for transition metal dichalcogenide-based electrochemical biosensors [142]. Although the transduction mechanism differs, this perspective broadens the discussion of how advanced sensing interfaces can be developed into clinically useful diagnostic systems.

The portable digital nanoparticle-enhanced plasmonic imaging system presented in Figure 7 illustrates this transition from an optical transducer to an integrated diagnostic platform [70]. In Figure 7a, the inflammatory biomarkers procalcitonin and C-reactive protein are captured from serum by antibodies immobilized on gold nanohole arrays and subsequently recognized by antibody-conjugated gold nanoparticles. Binding of these nanoparticles locally perturbs the extraordinary optical transmission of the nanohole array, producing a measurable optical contrast. Figure 7b shows the portable reader, which uses narrowband LED illumination and a CMOS camera to acquire wide-field transmission images without a tunable laser or conventional spectrometer. The electron micrograph in Figure 7c shows nanoparticles bound within or near the nanoholes, while Figure 7d presents the localized transmission contrast used for digital particle counting [70]. Automated low-volume deposition of capture antibodies enables microarray functionalization, as illustrated in Figure 7e, whereas Figure 7f shows the wafer-scale-fabricated plasmonic sensor chip. Finally, Figure 7g presents the patterned sensing fields and integrated QR identifiers used to associate individual sensor regions with fabrication and assay information [70]. Collectively, these elements demonstrate the integration of serum-compatible molecular recognition, multiplexed functionalization, scalable fabrication, portable optical interrogation, and automated sensor traceability. The platform was evaluated using serum from patients with sepsis, individuals with noninfectious systemic inflammatory response syndrome, and healthy controls, providing an important example of complete-system testing in clinically relevant samples [70]. Within the proposed translation framework, this study demonstrates Stage 3 system integration and initial Stage 4 real-sample validation; the reported evidence does not establish Stage 7 routine clinical deployment.

Clinical translation nevertheless requires validation beyond an initial single-center demonstration. Studies should include sufficiently large and representative patient cohorts, standardized preanalytical procedures, predefined decision thresholds, and direct comparison with accepted reference methods. Diagnostic sensitivity, specificity, predictive values, reproducibility, invalid test rates, and robustness to patient- and operator-dependent variation must be established. Multicenter evaluation is particularly important for confirming performance across populations, instruments, clinical sites, and sample-handling practices [143]. For POC in vitro diagnostics, the applicable FDA pathway or EU conformity assessment under the IVDR depends on intended use and device classification [144]. Clinical evidence generation, intended-user studies, and regulatory assessment can become major bottlenecks between prototype demonstration and market access.

### 5.2. Environmental and Food-Safety Monitoring

Environmental photonic sensors can target pathogens, heavy metals, pesticides, toxins, nutrients, gases, microplastics, and organic pollutants [95,145,146]. Their principal translational advantage is the potential to perform measurements directly at the sampling location and to provide spatially or temporally resolved information that cannot be obtained through intermittent laboratory analysis. The required performance nevertheless depends on the intended purpose, including regulatory compliance, early-warning surveillance, contamination-source identification, or continuous monitoring.

Environmental matrices introduce substantial analytical variability. Turbidity, salinity, pH, temperature, suspended particles, dissolved organic matter, and chemically related interferents can affect optical propagation, molecular recognition, and baseline stability [147,148,149]. Prolonged exposure may additionally cause biofouling, surface contamination, or degradation of functional coatings. Reliable operation therefore requires matrix-compatible sample handling, reference channels, antifouling strategies, temperature compensation, and signal-processing methods capable of distinguishing target-dependent responses from environmental drift. For particulate contaminants such as microplastics, quantitative analysis is further complicated by variations in particle size, morphology, polymer composition, concentration, and environmental weathering [150].

Translation from an optical transducer to a deployable monitoring tool requires coordinated integration of sample handling, microfluidics, recognition chemistry, optical interrogation, temperature regulation, electronics, and data processing. Knoben et al. demonstrated this system-level approach using a SiN interferometric platform developed for detecting pathogen-specific DNA relevant to aquaculture monitoring [151]. As illustrated in Figure 8, Figure 8a,b shows the wafer-level transfer and flip-chip bonding of GaAs photodetector arrays, while Figure 8c demonstrates the simultaneous population of multiple photonic dies with vertical-cavity surface-emitting lasers, photodetectors, and temperature sensors. A completely assembled and wire-bonded hybrid photonic chip is presented in Figure 8d, whereas Figure 8e,f show its backside resistive heater and front-side optical architecture, respectively. The latter contains an 850 nm laser, waveguide splitters, six asymmetric Mach–Zehnder interferometric biosensors, two auxiliary optical channels, and eight integrated photodetectors within a 3 × 5 mm^2^ footprint. The deposition of electrically conductive adhesive and subsequent placement and encapsulation of the chip within the printed-circuit-board cavity are shown in Figure 8g,h. Finally, Figure 8i presents the assembled microfluidic cartridge, which provides sample and reagent storage, fluid delivery, liquid-level monitoring, heating, electrical interfacing, and waste containment [151].

Collectively, Figure 8 demonstrates the progression from wafer-level component integration to a replaceable analytical cartridge combining optical excitation, detection, thermal control, selective functionalization, and automated fluid handling. Detection of an Aeromonas salmonicida-specific DNA sequence confirmed the biosensing functionality of the integrated platform, although validation using field-derived samples and extended autonomous operation remains necessary [151]. This example therefore establishes Stage 3 integration, while progression through real-sample validation and field evaluation remains necessary before Stage 7 deployment can be claimed.

Food-safety monitoring presents comparable challenges because food matrices are frequently heterogeneous, opaque, viscous, and chemically complex [19,152,153]. Photonic systems may enable rapid screening for pathogens, toxins, allergens, adulterants, spoilage indicators, and chemical residues across production and supply chains. Representative sampling and matrix-compatible preparation remain essential, however, because contamination may be spatially nonuniform and matrix components can interfere with both recognition and optical transduction. For routine environmental and food-safety monitoring, robustness, rapid analysis, low maintenance, reproducible calibration, and affordable cost per test may therefore be more important than an exceptionally low detection limit obtained under controlled laboratory conditions. For regulatory environmental monitoring, commercialization additionally depends on acceptance of the measurement method, including reference-method comparison and, where applicable, certification through schemes such as the Environment Agency’s Monitoring Certification Scheme (MCERTS) in England. Laboratory and field qualification can therefore determine deployment timelines; suitability for preliminary screening does not establish acceptance for compliance reporting [154].

### 5.3. Industrial and Structural Monitoring

Industrial photonic sensors can measure temperature, pressure, strain, vibration, chemical composition, and gas leakage, while structural monitoring applications include bridges, buildings, pipelines, aircraft, wind turbines, railways, and energy infrastructure [114,155,156,157,158]. These systems often detect physical parameters directly and therefore avoid some of the surface-chemistry and sample-handling challenges associated with biosensing. This partly explains why photonic sensing has achieved greater maturity in selected industrial applications. Deployment conditions can nevertheless be severe. Sensors may encounter high temperatures, ionizing radiation, electromagnetic interference, mechanical shock, corrosion, pressure cycling, or explosive atmospheres. Each component must withstand its local operating conditions, while remote interrogation can protect sensitive electronics from the harsh sensing environment. Long-term drift, hysteresis, fatigue, calibration stability, and failure probability are often more important than maximum sensitivity. These requirements become particularly important when sensors are embedded in structures or installed at inaccessible locations [159].

Fiber-optic technologies are well suited to remote, multiplexed, and distributed measurements because numerous sensing points can be interrogated through limited optical infrastructure. Integrated photonic sensors may provide complementary advantages where miniaturization, spectral selectivity, or multi-parameter detection is required. In both cases, raw optical data must be converted into actionable information, such as leak localization, identification of abnormal strain, estimation of structural degradation, or initiation of a process-control response. This requires integration with established industrial control, maintenance, and safety systems. A representative implementation is presented in Figure 9, where distributed optical fibers installed along a 1 km section of an operational high-speed railway bridge provided spatially resolved strain measurements [159]. Measurements from fibers attached to different regions of the bridge girders were combined with a mechanical model and temperature compensation to calculate axial, horizontal, and vertical displacements. The resulting information was integrated into a supervisory control and data acquisition system, thereby connecting distributed optical measurements with structural assessment and safety management [159].

The upper part of Figure 9 displays the complete monitored bridge section, with the spatial distribution of the measured response represented by a color scale. This overview allows the location of strain concentrations or anomalous structural behavior to be identified across the monitored distance. The lower part provides an enlarged view of a selected bridge section together with the corresponding axial strain and calculated axial, horizontal, and vertical displacements. Warning and inspection thresholds derived from structural safety requirements are also incorporated into the interface. Importantly, the same monitoring system enabled rapid comparison of strain distributions before and after two earthquakes, demonstrating how photonic measurements can support timely assessment of structural integrity following disruptive events [159]. This installation demonstrates field operation consistent with Stage 5. Although its integration into an operational monitoring workflow indicates deployment maturity, the cited case alone does not document the manufacturing and commercial evidence required to establish the complete Stage 7 pathway.

Industrial qualification depends on the installation and safety function, with ATEX requirements relevant to potentially explosive atmospheres and IEC 61508 or sector-specific standards relevant to functional safety [160]. Commercialization bottlenecks consequently centre on installation qualification, endurance testing, and safety assurance, whose scope differs substantially between advisory monitoring and automatic protective systems. Industrial adoption is most likely when photonic sensors provide a clear advantage over electrical alternatives, including immunity to electromagnetic interference, electrical passivity, remote interrogation, high-temperature operation, or distributed measurement over long distances [1,24]. Translation should therefore prioritize applications in which these characteristics reduce operational risk, maintenance requirements, or lifecycle cost. Across all three application pathways, successful deployment depends not on achieving the highest isolated device performance, but on delivering reliable information within the technical, economic, and operational constraints of the intended application.

### 5.4. Commercial Deployment and Lessons from Established Products

Biacore surface plasmon resonance instruments illustrate Stage 7 commercial deployment in biopharmaceutical analysis and quality control. Established applications include active concentration measurements, target-binding analysis, and assessment of drug potency and stability [161]. Their commercial role is distinct from routine patient diagnosis: the measurement supports decisions about therapeutic molecules and product quality. This case suggests that a key enabler of translation is the development of assays that connect optical binding responses to specific pharmaceutical questions. The resulting value comes from interpreting molecular activity within an established analytical workflow, allowing a laboratory-based instrument to achieve commercial utility without requiring point-of-care operation.

A contrasting example is the distributed fiber-optic monitoring system commissioned for the Trans-Anatolian Natural Gas Pipeline. According to Luna’s commissioning announcement, the installation covers the 1811 km route and supports leak detection, pipeline security, and facility protection. Commissioning was completed in November 2023 following a six-year project involving Luna, ABB, and the pipeline operator [162]. This provides documented evidence of Stage 7 deployment at infrastructure scale. The case illustrates how collaboration between the sensing supplier, automation contractor, and asset owner can connect distributed optical measurements to installation requirements and operator-facing warnings. Its practical value lies in locating events across an extensive asset, while delivering outputs that the operator can use without interpreting the underlying optical data.

These cases reveal two distinct routes across the deployment threshold: embedding molecular measurements within established laboratory decision processes and embedding spatially distributed measurements within infrastructure operations. They suggest that application-specific interpretation and a workable delivery model are central enabling factors. Commercial deployment does not necessarily require eliminating laboratory instrumentation or minimizing every component cost; it requires a complete system whose information justifies its operational burden within the intended use.

## 6. The Laboratory-to-Field Performance Gap

The transition from a laboratory prototype to a deployable photonic sensor introduces sources of uncertainty that are often absent or deliberately minimized during device characterization. As summarized in Figure 10, differences in sample composition, environmental conditions, manufacturing variability, operational duration, and validation methodology can progressively alter the optical baseline, target response, calibration, and failure probability. Laboratory performance can therefore be preserved in practice only when these translation-induced effects are identified, quantified, and controlled.

### 6.1. Simplified Samples Versus Real Matrices

Laboratory characterization commonly employs purified analytes and buffered solutions under controlled physicochemical conditions. Real clinical, environmental, food, and industrial samples contain interferents, particulates, macromolecules, cells, and variable concentrations of salts and organic compounds [163]. These constituents can produce nonspecific adsorption, competitive binding, optical absorption or scattering, surface fouling, and bulk refractive-index changes that are unrelated to the target. They may also alter receptor activity and analyte-transport kinetics [164]. Consequently, calibration established in buffer cannot be assumed to remain valid in serum, wastewater, food extracts, or process fluids. The magnitude of this matrix-induced performance loss must be established using representative samples and validated against an accepted reference method.

### 6.2. Controlled Conditions Versus Variable Environments

Outside the laboratory, variations in temperature, humidity, pressure, mechanical stress, vibration, contamination, optical alignment, and sample delivery can perturb the measured signal independently of the target [29]. Resonant and interferometric architectures are particularly vulnerable because small changes in refractive index, optical path length, coupling, or polarization can generate responses comparable to the target-induced signal. Reference channels, differential interrogation, thermal compensation, robust packaging, and automated fluidic control can reduce these disturbances, but their effectiveness must be demonstrated across the complete environmental envelope of the intended application [67].

### 6.3. Optimized Devices Versus Manufacturing Variability

Exceptional performance from an individually optimized device does not establish manufacturing reproducibility. Lithographic and etching tolerances, sidewall roughness, film-thickness variation, material inhomogeneity, coupling-gap deviations, bonding errors, and functionalization nonuniformity can alter propagation loss, spectral position, linewidth, extinction ratio, and sensing response [165,166]. Resonant and slow-light structures are especially sensitive to dimensional deviations because their performance depends strongly on phase matching, dispersion, and optical coupling. Translation therefore requires statistical evaluation across independently fabricated devices, wafers, batches, and functionalization runs. Tolerance-aware design, process monitoring, post-fabrication trimming, reference structures, and transferable calibration strategies may be required to maintain an acceptable production yield [167].

### 6.4. Short-Term Demonstrations Versus Long-Term Operation

Short-duration measurements do not reveal degradation occurring during storage, transport, regeneration, repeated use, or continuous operation [139]. Photobleaching, receptor denaturation, coating desorption, biofouling, corrosion, mechanical fatigue, adhesive creep, and drift of optical or electronic components can progressively modify both the baseline and target response. Regeneration may additionally reduce binding capacity when it incompletely removes the analyte or damages the recognition interface [168]. Operational durability should therefore be evaluated through baseline drift, signal retention, calibration stability, regeneration efficiency, shelf life, and performance following application-relevant thermal, chemical, humidity, and mechanical stress [168].

### 6.5. Publication Metrics Versus Application-Relevant Evidence

Figure 10 illustrates why device-level performance alone cannot establish deployment readiness. A photonic sensor may show a large resonance shift, narrow linewidth, or low nominal detection limit under controlled conditions, yet still fail to provide a reliable application-level measurement when the response is affected by sample variability, environmental drift, packaging instability, or device-to-device variation. The critical question is therefore not whether the optical response is measurable, but whether it remains sufficiently accurate and reproducible for the intended decision after these sources of variation are introduced.

The contrast between mature fiber-optic monitoring systems and emerging chip-scale biosensors is particularly instructive. Fiber-optic sensing has achieved broad industrial use because its long-distance, passive, and multiplexed measurement capabilities address operational needs that conventional electrical systems cannot readily meet; its interrogation hardware, installation methods, calibration practices, and reliability qualification have also matured together. By comparison, integrated biosensors and plasmonic or metasurface platforms often demonstrate strong intrinsic optical performance, but translation remains constrained by assay reproducibility, complex sample matrices, surface stability, and the need for compact, robust, and economical readout. Thus, Figure 10 should be interpreted as a transition from device metrics to evidence-based system validation, rather than as another general list of desirable performance characteristics.

## 7. Integration as the Central Translational Challenge

The performance of a deployable photonic sensor is determined by the compatibility of its optical, fluidic, chemical, electronic, and mechanical subsystems rather than by the isolated performance of the photonic transducer [50]. Integration modifies optical loss, noise, thermal response, analyte transport, calibration stability, and the failure modes of the complete system. Interfaces between components are particularly critical: packaging can alter optical coupling and thermal behavior, microfluidics can introduce bubbles or mechanical stress, and surface modification can increase propagation loss or shift the operating point of a resonant device. Translation therefore requires concurrent engineering supported by optical-power, noise, thermal, fluidic, and uncertainty budgets. The objective is to preserve application-relevant analytical performance while reducing alignment sensitivity, operator dependence, measurement complexity, and total cost.

Light-source and detector integration must be matched to the selected interrogation method and required measurement uncertainty. Narrow-linewidth tunable lasers enable high-resolution tracking of resonant and interferometric features but require accurate wavelength calibration and stabilization of drive current and temperature [169,170]. LEDs and broadband emitters provide more economical and potentially more robust illumination, although their spectral power density and wavelength stability may restrict measurement resolution [171]. Photodiodes support compact intensity-based detection, whereas detector arrays or miniature spectrometers are required when analytical information is distributed across a broader spectral interval. Spectrometer-free approaches, including resonance locking, fixed-wavelength interrogation, differential intensity measurement, optical filtering, and wavelength-to-intensity conversion, can substantially reduce system size and cost [172]. However, these strategies transfer performance requirements to source stability, detector linearity, reference-channel accuracy, and calibration. Hybrid or heterogeneous integration of III–V light sources, silicon or silicon-nitride photonic circuits, and photodetectors can reduce alignment-sensitive interfaces, but thermal crosstalk, electrical isolation, material incompatibility, interlayer coupling loss, fabrication yield, and repairability must be considered at the system-design stage [173].

Optical coupling and packaging frequently represent dominant sources of insertion loss and measurement variability. Grating couplers permit vertical fiber attachment and wafer-level testing but exhibit limited bandwidth, polarization dependence, and sensitivity to fiber position, illumination angle, fabrication tolerances, and changes in the surrounding refractive index [113]. Edge couplers can provide lower loss and broader bandwidth but require accurately prepared facets and stable in-plane alignment [174]. Permanent fiber attachment improves mechanical robustness while introducing possible errors associated with adhesive shrinkage, thermal-expansion mismatch, connector repeatability, and long-term creep. Free-space coupling is attractive for imaging-based and metasurface sensors but remains vulnerable to vibration, contamination, illumination nonuniformity, and angular misalignment. Packaging must stabilize these optical interfaces while simultaneously providing fluidic sealing, electrical access, thermal management, mechanical protection, and controlled exposure of the active sensing region. Encapsulation materials should not introduce excessive absorption, stress-induced birefringence, outgassing, or chemical contamination. Packaging performance should consequently be assessed using distributions of coupling loss, alignment-tolerance measurements, leakage tests, thermal cycling, vibration, humidity exposure, and accelerated aging rather than by initial insertion loss alone [171].

For chemical and biological sensors, microfluidic integration determines the conditions under which the target reaches and interacts with the recognition surface [127,175]. The measured response depends on convection, diffusion, receptor kinetics, surface accessibility, and local analyte depletion. High optical sensitivity cannot compensate for inefficient analyte transport or inadequate sample preparation. Channel geometry, flow rate, mixing, sample volume, residence time, and washing conditions must therefore be co-designed with receptor affinity and the spatial distribution of the optical sensing field. Real samples may additionally require filtration, separation of interfering components, target enrichment, controlled dilution, reagent mixing, incubation, washing, and waste isolation. Each operation can cause analyte loss, contamination, dead volume, carryover, or an increase in the total sample-to-result time. Bubble formation is particularly detrimental because it disrupts flow, changes the local refractive index, scatters light, and generates large transient signals. Adsorption to tubing and channel walls may also reduce analyte recovery, especially at low target concentrations. The complete fluidic subsystem should therefore be evaluated through recovery efficiency, carryover, pressure stability, leakage, clogging probability, reagent consumption, and processing time. Disposable cartridges can isolate contaminated fluid paths from reusable optical and electronic components, provided that cartridge positioning, optical registration, and fluidic sealing remain sufficiently reproducible to preserve calibration. A representative system-level implementation integrated a Si_3_N_4_ chirped guided-mode-resonance sensor with membrane-based blood separation, gravity-assisted plasma delivery, capillary pumping, reference sensing and waste containment in a disposable cartridge compatible with a compact optical reader (Figure 11a–e) [176]. This architecture illustrates how sample preparation, fluid transport, differential photonic detection and waste handling can be co-designed within a unified point-of-care measurement chain.

Surface functionalization converts an optically responsive device into a target-selective analytical system, but it can also introduce substantial variability [177]. Differences in surface activation, receptor density, molecular orientation, accessibility, and retained biological activity influence binding capacity and apparent affinity. Random immobilization may obstruct recognition sites or produce heterogeneous receptor populations, whereas oriented or site-specific attachment can improve effective activity and reduce batch-to-batch variation. The recognition layer must remain sufficiently uniform to preserve optical mode overlap without introducing excessive scattering, absorption, or nonspecific adsorption. The measured assay response is therefore governed by a coupled sequence of mass transport, interfacial recognition, and optical transduction rather than by receptor immobilization alone. As summarized in Figure 12, analyte delivery to the sensor surface may be affected by bubbles, reagent depletion or contamination, flow instability and fluidic-channel misalignment (Figure 12a) [28]. The subsequent binding response depends on bioreceptor orientation and surface density, receptor stability, reagent variability and nonspecific or off-target adsorption (Figure 12b) [28]. Finally, conversion of the surface refractive-index perturbation into a measurable resonance shift is influenced by fabrication tolerances, waveguide wetting, oxide-opening variability and surface contamination (Figure 12c) [28]. These coupled contributions demonstrate that functionalization reproducibility must be evaluated together with the microfluidic environment and the intrinsic surface response of the photonic transducer.

Before packaging, functionalization quality control should distinguish total receptor coverage from accessible binding-site density [178]. The latter can be estimated using calibrated target-binding measurements at verified saturation, corrected for nonspecific binding; reporting active receptor molecules per square micrometer additionally requires a validated binding stoichiometry. Recommended metrics include mean accessible binding-site density (sites per µm^2^), measurement uncertainty, within-wafer and between-wafer coefficients of variation, and the fraction of sampled sites meeting predefined acceptance limits [28]. Testing should use spatially distributed sacrificial sites representative of the sensing surfaces and processed identically across batches. Acceptance limits should be linked to assay performance rather than universal density thresholds.

Storage stability must be verified under relevant temperature, humidity, packaging, and transport conditions rather than only immediately after immobilization [179]. Regeneration can reduce cost in reusable systems, but repeated chemical treatment may denature receptors, damage antifouling coatings, or progressively shift the optical baseline. The choice between disposable and regenerable sensing interfaces should therefore consider receptor lifetime, contamination risk, assay frequency, calibration stability, and cost per valid measurement. Microarray spotting, inkjet deposition, photochemical patterning, and microfluidic functionalization may support scalable multiplexing, provided that deposition uniformity, spatial registration, and cross-contamination are adequately controlled.

Electronics and data processing must coordinate optical excitation, detector biasing, temperature regulation, fluidic actuation, signal amplification, digitization, timing, and communication. Electronic noise, quantization error, detector saturation, limited sampling rate, and synchronization errors can directly restrict measurement precision. Automated control should therefore be designed according to the dynamic range and temporal response of the sensor [180]. Built-in diagnostic functions can identify insufficient optical power, detector saturation, abnormal fluid flow, temperature excursions, reference-channel failure, or measurements outside the validated operating range. Conversion of the raw optical signal into an estimate of the measurand may require baseline subtraction, spectral normalization, resonance fitting, wavelength or phase tracking, drift correction, kinetic analysis, and application of a calibration model. These algorithms should propagate measurement uncertainty and reject invalid measurements rather than generate unconstrained estimates. Wireless communication and user interfaces can facilitate portable or distributed operation, but data integrity, cybersecurity, latency, power consumption, and compatibility with existing information systems must also be addressed [181]. Data-assisted methods may improve sensor fusion, spectral interpretation, drift correction, and anomaly detection, but they require representative training data, independent validation, uncertainty estimation, and evaluation under device, sample, and environmental distribution shifts [182,183]. Computational processing should complement physically stable sensing and traceable calibration rather than compensate for unreliable hardware.

Reference channels and internal standards provide an important connection between system integration and measurement traceability. A reference may comprise an unfunctionalized photonic structure, a surface modified with a nonbinding receptor, an internal optical standard, or an auxiliary sensor monitoring temperature, pressure, source power, or bulk refractive index. Differential sensing can suppress common-mode disturbances only when the sensing and reference channels exhibit sufficiently matched optical, thermal, fluidic, and surface responses. Poorly matched references can introduce bias or inadvertently remove part of the target-specific signal. Self-referenced architectures based on multiple wavelengths, modes, resonances, polarizations, or spatial channels can compensate for source fluctuations, coupling changes, environmental drift, and nonspecific adsorption [184]. Temperature compensation may similarly combine an optical reference with an independently calibrated temperature sensor. Nevertheless, referencing does not eliminate the need for calibration. Quantitative operation requires traceable standards, validated calibration models, defined recalibration intervals, and objective criteria for identifying calibration failure. Calibration transfer among devices is reliable only when fabrication and functionalization variability are adequately represented by the calibration model.

Integration should therefore be treated as a primary design dimension rather than as a final assembly step applied to an optimized photonic device. Decisions concerning the transducer, source, detector, optical interface, fluidics, recognition chemistry, packaging, electronics, referencing, and data processing are strongly interdependent and should be made within a unified system architecture [126]. A successful integrated platform is not necessarily the one with the lowest component-level detection limit, but the one that maintains traceable and reproducible performance after assembly, storage, transport, sample processing, and operation by the intended user. This shift from component integration to measurement-chain co-design is essential for converting advanced photonic structures into reliable sensing technologies.

## 8. Manufacturing, Standardization, and Commercialization

The transition from a validated prototype to a commercial photonic sensor requires controlled industrialization rather than further optimization of isolated device performance. The central objective becomes the reproducible manufacture of systems that remain within predefined specifications at an acceptable yield, cost, and failure rate. CMOS-compatible platforms offer access to wafer-scale lithography, deposition, etching, metrology, and automated handling, but process compatibility alone does not establish production readiness [47,185,186]. Exposed sensing regions, nonstandard waveguide geometries, heterogeneous materials, protective coatings, and post-fabrication modification can introduce operations that are not included in conventional foundry workflows. Manufacturing feasibility must therefore be demonstrated through statistically meaningful wafer maps and independent production lots, with critical parameters monitored from incoming materials to final system testing. The relevant outcome is not the performance of the best device, but the proportion of manufactured systems that satisfy the complete product specification without exceptional adjustment.

Foundry access and process design kits can reduce development time by providing qualified materials, component libraries, layout rules, compact models, and standardized test structures. For sensing applications, however, conventional telecommunications-oriented kits require additional information describing exposed waveguides, analyte-access windows, environmental interaction, functional-layer deposition, and the statistical effects of dimensional variability on sensing performance [137]. These data should support tolerance-aware design in which acceptable operating margins are maintained across the expected fabrication distribution. Designs positioned close to critical coupling, modal cutoff, exceptional points, or other sharply varying operating conditions may provide superior nominal performance while exhibiting poor production yield. Manufacturability therefore requires optimization of the performance distribution rather than its maximum value. Where residual variability cannot be eliminated economically, post-fabrication trimming, reference structures, calibration transfer, or adjustable interrogation may be preferable to imposing narrower process tolerances.

A scalable manufacturing route must incorporate rapid testing at intermediate production stages. Manual characterization using tunable laboratory lasers, polarization controllers, optical spectrum analyzers, and actively aligned fibers is poorly suited to volume manufacturing because test time and skilled labor may exceed the cost of chip fabrication [187]. Wafer-level measurements should identify propagation loss, coupling efficiency, resonance position, detector response, and reference-channel functionality before expensive downstream processing. Known-good-die selection can prevent defective devices from entering assembly, coating, or functionalization stages, whereas in-line metrology can detect systematic process drift before an entire production lot is completed [188]. The resulting data should be connected to a manufacturing control plan that defines critical parameters, sampling frequency, acceptance limits, corrective actions, and criteria for lot release. This approach converts fabrication variability from an uncontrolled source of uncertainty into a measurable component of production quality.

Packaging must similarly be addressed as a manufacturing operation with quantifiable yield, cycle time, and reliability rather than solely as a technical interface. Assembly may dominate unit cost when optical alignment, fluidic sealing, electrical interconnection, environmental protection, and mechanical fixation require separate manual procedures. Commercially viable packages should minimize alignment degrees of freedom, use standardized interfaces where possible, and remain compatible with automated placement, bonding, sealing, and inspection [189]. Package qualification must be performed on the assembled product because failures often originate from interactions among otherwise acceptable components. Temperature cycling, humidity, vibration, shock, pressure, connector use, cleaning, transport, and application-specific chemical exposure can reveal adhesive creep, seal degradation, stress-induced spectral changes, contamination, or loss of optical alignment. Accelerated tests should be related to credible use conditions through defined failure mechanisms rather than used only as empirical demonstrations of durability.

Standardization at this stage should convert the analytical and reliability criteria established earlier in the development process into executable product specifications. Each specification requires a defined test method, traceable reference, acceptance limit, and measurement uncertainty. Harmonization is particularly important for quantities whose reported values depend on acquisition settings or statistical treatment. Instead of introducing additional performance metrics, commercialization requires agreement on how existing metrics are verified during design qualification, production release, and periodic quality control [190]. Test procedures should define the measurand range, environmental conditions, instrumentation, data-processing version, calibration hierarchy, number of devices and production lots, treatment of failed measurements, and rules for exclusion or recalibration. Interlaboratory studies are valuable because they reveal whether a method remains transferable when instruments, operators, sample preparation, and production origin change. Standardized reporting should also distinguish design verification, which establishes that engineering specifications have been met, from application validation, which establishes that the product performs its intended function.

Benchmarking should serve as a formal product-development decision rather than as a general comparison of sensing principles. The appropriate comparator is the established technology used for the same operational decision, evaluated with identical samples, environmental conditions, decision thresholds, and definitions of error. A photonic sensor need not exceed the incumbent method in every analytical metric. Its commercial advantage may instead arise from faster measurement, higher information density, continuous or distributed monitoring, remote interrogation, electrical passivity, reduced sample volume, or operation where conventional instrumentation is impractical. Conversely, a modest analytical improvement may not justify additional optical hardware, calibration, or workflow modification. Benchmarking should therefore identify the minimum performance advantage required for adoption and determine whether that advantage is maintained after manufacturing variation, maintenance, operator time, and unsuccessful measurements are included [191].

Commercial viability must be assessed using the cost per valid and decision-relevant measurement rather than the price of the photonic chip. A complete economic model should include fabrication yield, packaging, quality control, functional materials, disposable components, reader instrumentation, calibration, installation, operator training, maintenance, data infrastructure, service life, failure replacement, and waste management [192]. Production volume should be considered explicitly because wafer fabrication, custom packaging, and regulatory qualification contain different fixed and variable cost components. Sensitivity analysis can identify whether profitability is most strongly limited by yield, assembly time, consumable cost, instrument lifetime, or market volume, thereby directing engineering effort toward economically consequential improvements. Commercial value may also include avoided costs, such as reduced equipment downtime, earlier detection of structural deterioration, fewer confirmatory analyses, or prevention of environmental release. These benefits must be demonstrated within the intended workflow rather than inferred from laboratory performance.

As a quantitative benchmark, assembly, optical packaging, and testing have been estimated to account for approximately 70–80% of the overall cost of a photonic package [193]. This general integrated-photonics estimate provides context for the resonant, interferometric, and waveguide-engineered platforms in Table 2, but does not establish separate packaging-to-chip ratios for these architectures. The remaining 20–30% cannot automatically be assigned entirely to the bare die. For fiber-optic platforms, the corresponding comparison concerns the unencapsulated sensing fiber and the packaged, installed sensor, with shared interrogation costs reported separately. For plasmonic and metasurface assays, disposable substrate and cartridge costs should likewise be separated from reusable reader costs. Reliable numerical comparisons therefore require matched production volumes, yields, and clearly defined cost boundaries.

For biochemical applications, suitable comparators include ELISA for photonic protein-concentration assays and PCR or RT-qPCR for photonic nucleic-acid assays, provided that the methods address the same measurand, sample matrix, and intended decision. Existing laboratory infrastructure can favor established methods: an ELISA workflow may share plate-reading and washing equipment across numerous assays, whereas adopting a photonic platform may require a dedicated reader. Conversely, an on-demand photonic cartridge may reduce batch-accumulation delays and the per-sample overhead of standards and controls in small ELISA runs. However, removable plate strips can reduce unused-well wastage, and photonic cartridges also require appropriate quality controls. These advantages must be evaluated against cartridge price, shelf life, and achievable throughput. Table 3 summarizes the principal differences relevant to the purchasing decision.

Reader utilization can change the economic conclusion even when consumable savings are demonstrated. In an illustrative scenario, an additional €10,000 investment in a photonic reader would be recovered after 5000 reportable results if the saving were €2 per result, assuming equivalent analytical performance and no other cost differences. If additional maintenance costs €1000 annually, annual net savings would equal €2 multiplied by the number of reportable results, minus €1000. At 2000 results per year, this would yield €3000 in annual net savings and a simple payback period of approximately 3.3 years, excluding financing and discounting. These values are hypothetical rather than market quotations and illustrate the dependence of economic viability on testing volume.

Regulatory and certification pathways should be established before design freeze because they determine evidence requirements, material selection, software controls, documentation, and permissible design changes. The applicable framework depends on the intended use: clinical systems require controlled manufacturing and evidence of analytical and clinical performance; environmental instruments must comply with relevant sampling, calibration, and reporting procedures; and industrial systems may require qualification for hazardous locations, electromagnetic compatibility, pressure containment, mechanical durability, or functional safety. Risk management should connect identified hazards to design controls, verification tests, residual-risk assessment, and post-market surveillance. Software used for calibration, signal interpretation, quality control, or decision generation must be version-controlled and validated together with the hardware because algorithmic changes can alter the reported result without modifying the sensor itself.

Successful commercialization therefore depends on maintaining a stable relationship among product specifications, manufacturing controls, validation evidence, and user requirements. Late changes to the photonic architecture, package, surface treatment, supplier, or signal-processing algorithm may invalidate earlier qualification and should be governed through formal change control. Supplier qualification, component traceability, lot genealogy, complaint analysis, and field-failure reporting provide the feedback needed to maintain performance after market introduction. Manufacturing and commercialization are consequently not downstream administrative stages but evidence-generating processes that determine whether laboratory performance can be reproduced economically, certified for a defined purpose, and sustained throughout the product lifecycle.

## 9. Future Directions: Moving Beyond Sensitivity

Data-assisted methods can support spectral interpretation, sensor fusion, drift correction, anomaly detection, and multidimensional classification. Their reliability requires representative datasets, independent external validation, uncertainty estimation, and evaluation under distribution shifts [194]. Artificial intelligence should therefore complement physically robust sensing and traceable calibration rather than compensate for unstable devices or inadequate experimental data [130,195]. Shared datasets, standardized reference samples, harmonized reporting protocols, and interlaboratory comparisons will be essential for establishing reproducibility and enabling objective benchmarking against established analytical methods. A promising implementation strategy combines a disposable functionalized cartridge with a reusable optical and electronic reader, thereby separating the contaminated or degradable sensing interface from the higher-cost instrumentation. Heterogeneous integration of photonics, electronics, microfluidics, functional materials, light sources, and detectors could further reduce system footprint, alignment sensitivity, and operator dependence [196]. As summarized in Figure 13, future progress should be evaluated through reproducibility, stability, manufacturability, traceability, and application-level benefit. Packaging, functionalization, sample handling, interrogation, and data processing should therefore be developed concurrently with the photonic transducer from the earliest design stage.

## 10. Conclusions

The transition from laboratory demonstration to real-life application remains a central challenge for emerging and transitional photonic sensing platforms, including many integrated biosensors, plasmonic sensors, metasurfaces, and waveguide-engineered devices. In contrast, mature fiber-optic technologies demonstrate that broad deployment is achievable when the sensing principle, interrogation hardware, installation method, reliability qualification, and application value have evolved together. For emerging platforms, sensitivity, quality factor, resonance linewidth, and nominal limit of detection describe the intrinsic response of the photonic element, but do not alone establish practical performance.

Translation should begin with a clearly defined application and the specific decision the measurements must support. The target range, sample matrix, operating environment, required response time, service life, acceptable uncertainty, workflow, and total cost should guide the selection of the sensing architecture and readout strategy. The most appropriate platform is therefore not necessarily the most intrinsically sensitive, but the one that provides sufficient analytical performance with a robust measurement chain and a clear advantage over the established method.

For emerging chemical and biosensing platforms, the decisive evidence extends beyond measurements in purified targets, controlled environments, and individually optimized prototypes. Performance should be validated in representative matrices and against relevant interferents, using independently fabricated devices and batches, multiple operators, and application-relevant operating timescales. Agreement with an established reference method, together with accuracy, precision, recovery, false-result rates, calibration stability, fouling resistance, response time, storage lifetime, and manufacturing yield, establishes whether the optical response can support a reliable practical decision.

Successful deployment further requires concurrent engineering of the photonic transducer, recognition interface, sample handling, source and detector, electronics, packaging, referencing, and data-analysis framework. Data-assisted analysis, heterogeneous integration, compact interrogation, and cartridge–reader architectures are valuable when they reduce measurement uncertainty, operational complexity, or cost per valid result. Progress should therefore be judged not by isolated record metrics, but by the realization of photonic sensing systems that are selective, reproducible, robust, traceable, manufacturable, economically viable, and demonstrably fit for their intended application.

## Figures and Tables

**Figure 1:**
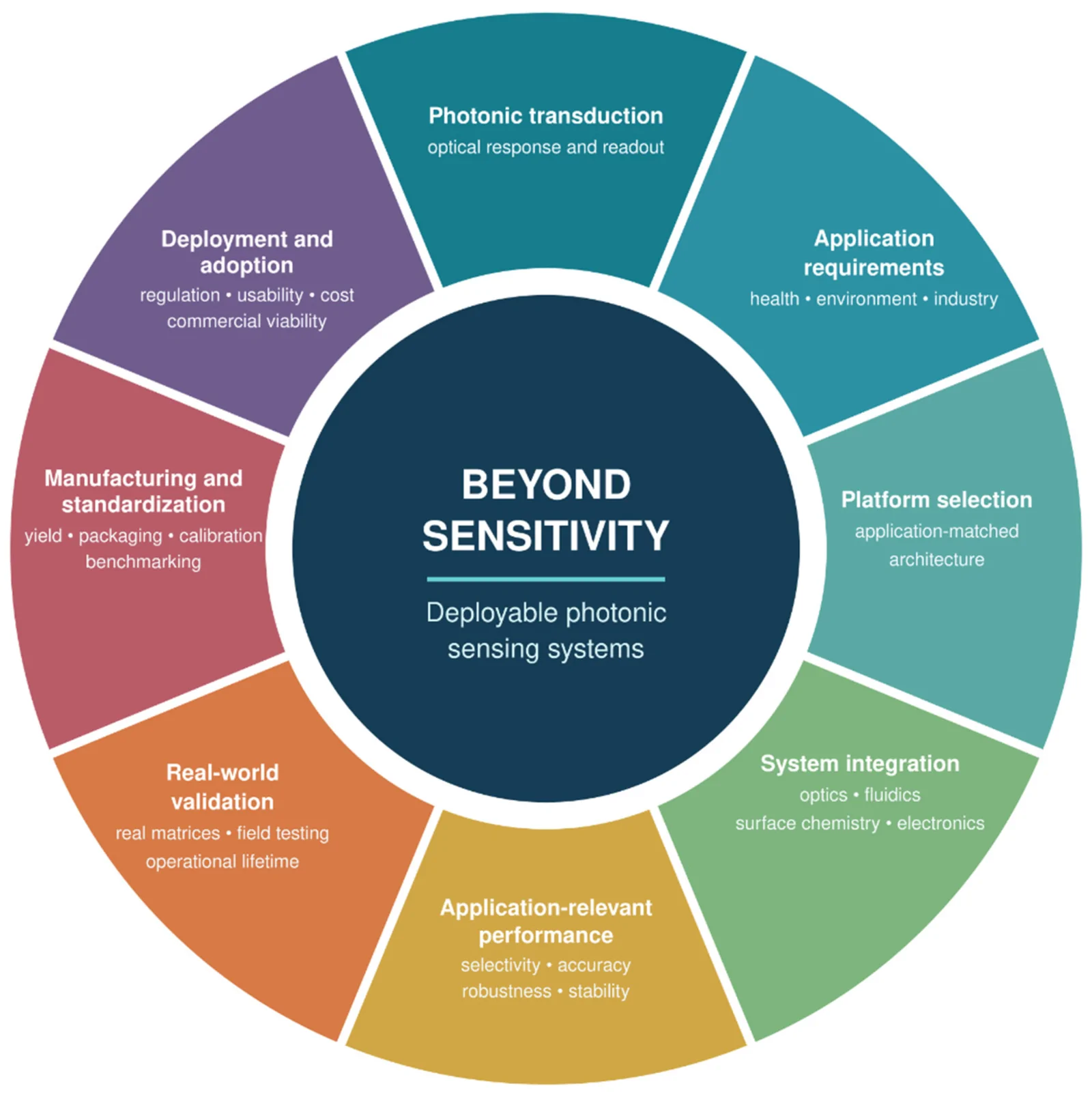
Application-driven framework for translating photonic sensing elements into deployable sensing systems beyond sensitivity.

**Figure 2:**
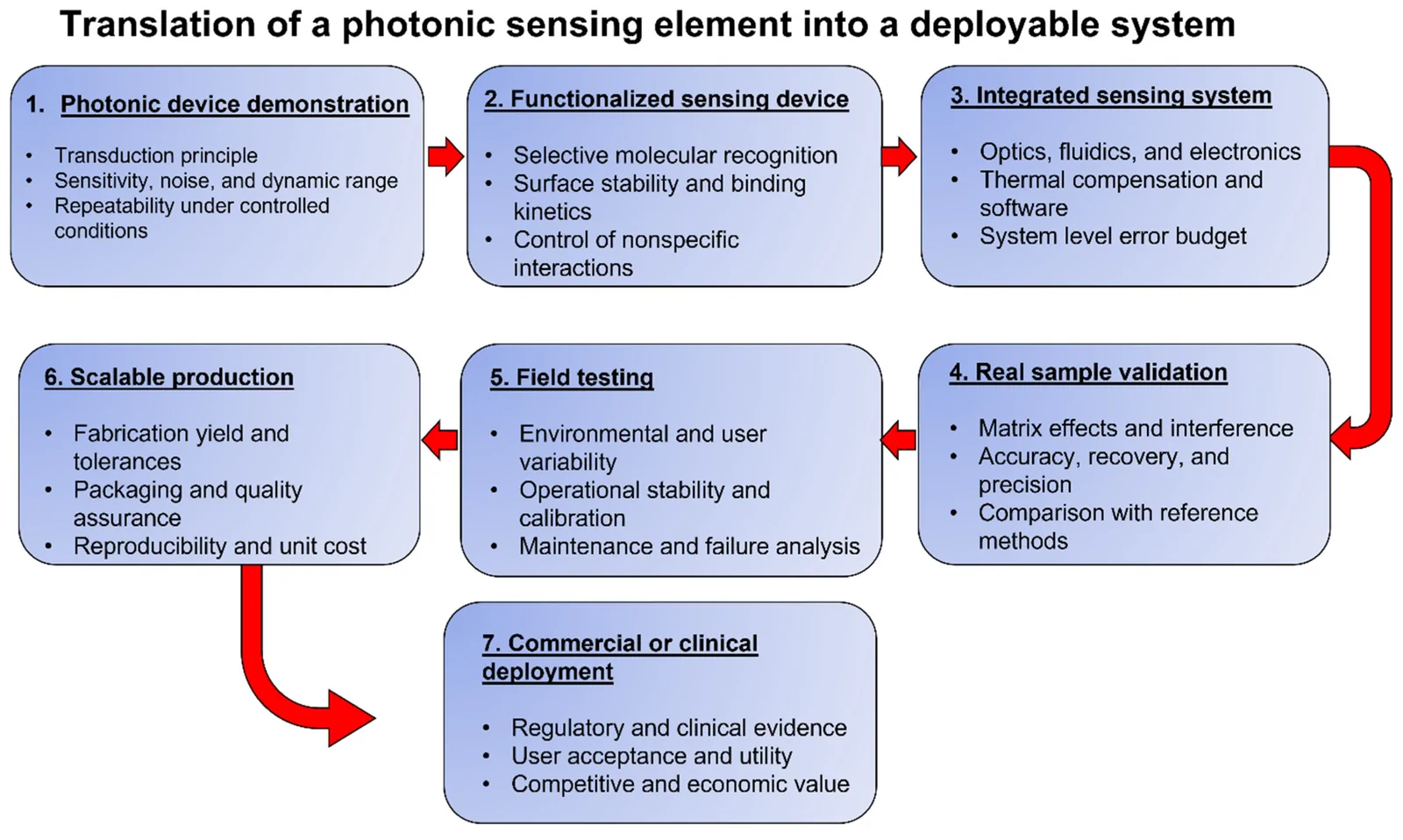
Conceptual seven-stage pathway for translating photonic sensing elements into deployable systems. The stages outline development and validation requirements rather than the proportion of technologies at each maturity level. Development is iterative, although only the forward progression is shown.

**Figure 3:**
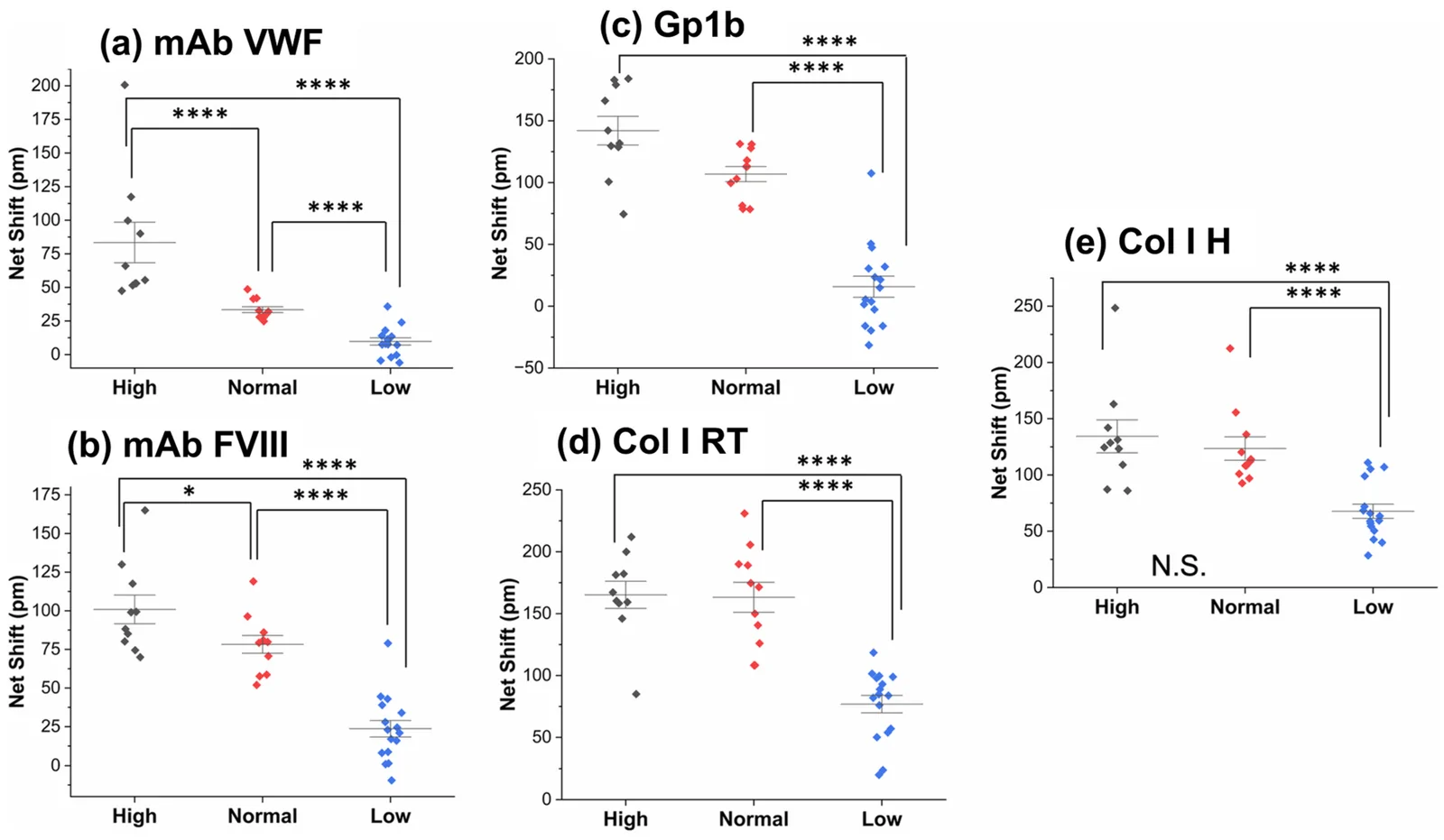
Assay responses from 37 single-donor plasma samples, previously characterized by reference-standard methods and classified as high, normal, or low VWF. The lower, central, and upper bars represent the 25th, 50th (median), and 75th percentiles, respectively. Statistical significance is denoted by * for 95% confidence and **** for 99.99% confidence. Responses were measured using immobilized: (**a**) anti-VWF monoclonal antibody (mAb); (**b**) anti-FVIII mAb; (**c**) Gp1b; (**d**) rat-tail collagen type I; and (**e**) human collagen type I [59].

**Figure 4:**
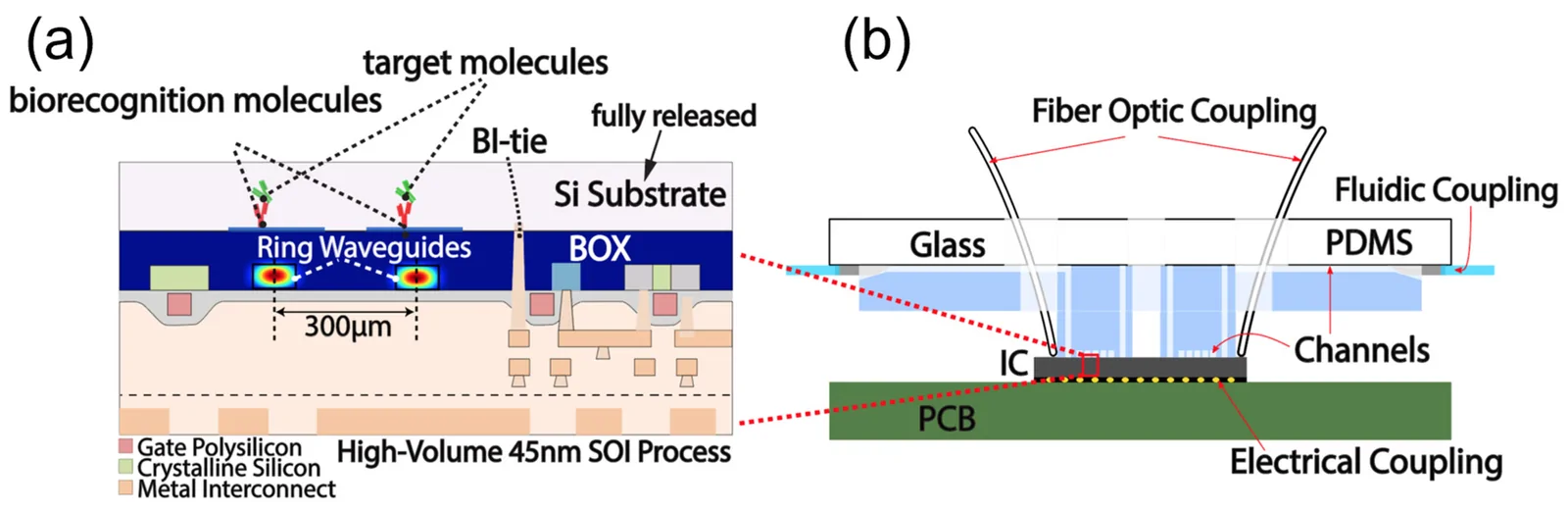
Multilayer packaging architecture for an integrated electronic–photonic biosensor. (**a**) Cross-section of the CMOS silicon-photonic platform showing backside substrate removal to expose the sensing region to fluidic samples. (**b**) Cross-section of the assembled package incorporating photonic ports for fiber coupling, multilayer fluidic routing, flip-chip electrical connections, and glass-supported mechanical stabilization [29].

**Figure 5:**
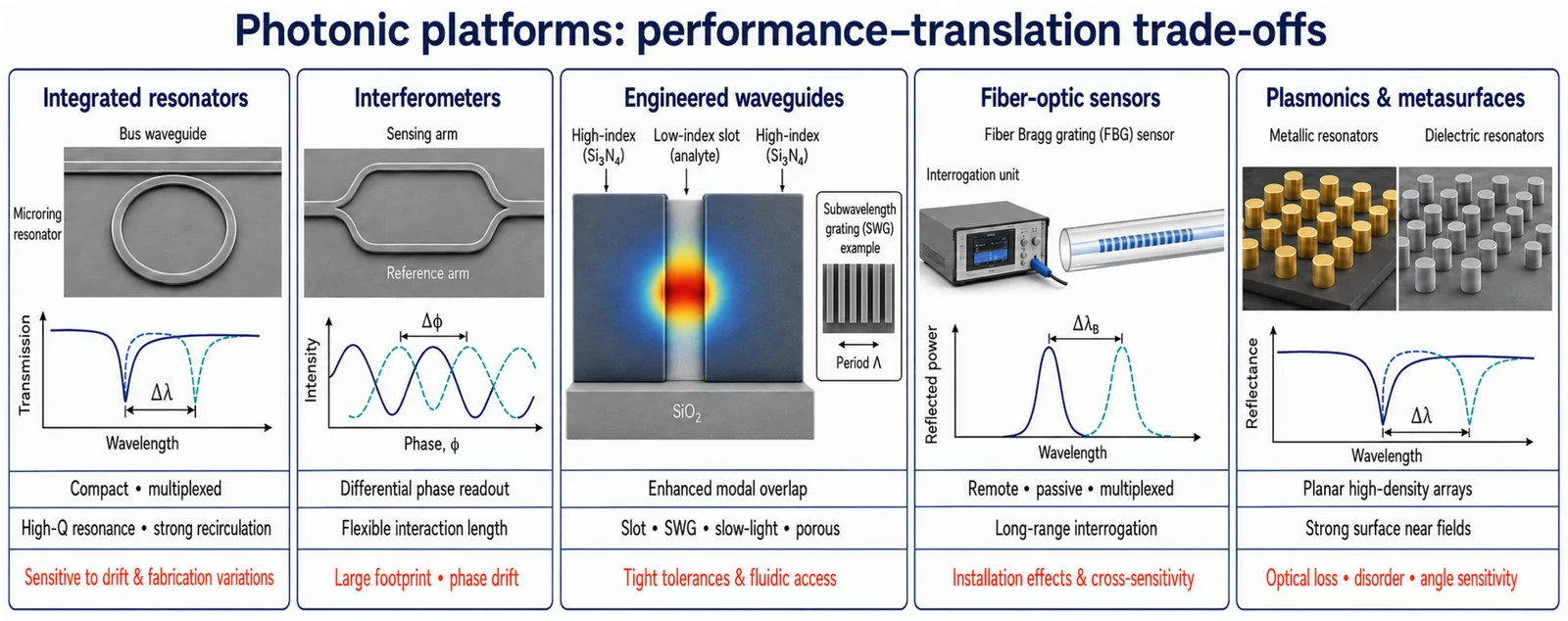
Schematic comparison of major photonic sensing platforms and their principal performance–translation trade-offs. The device illustrations are conceptual and not drawn to scale.

**Figure 6:**
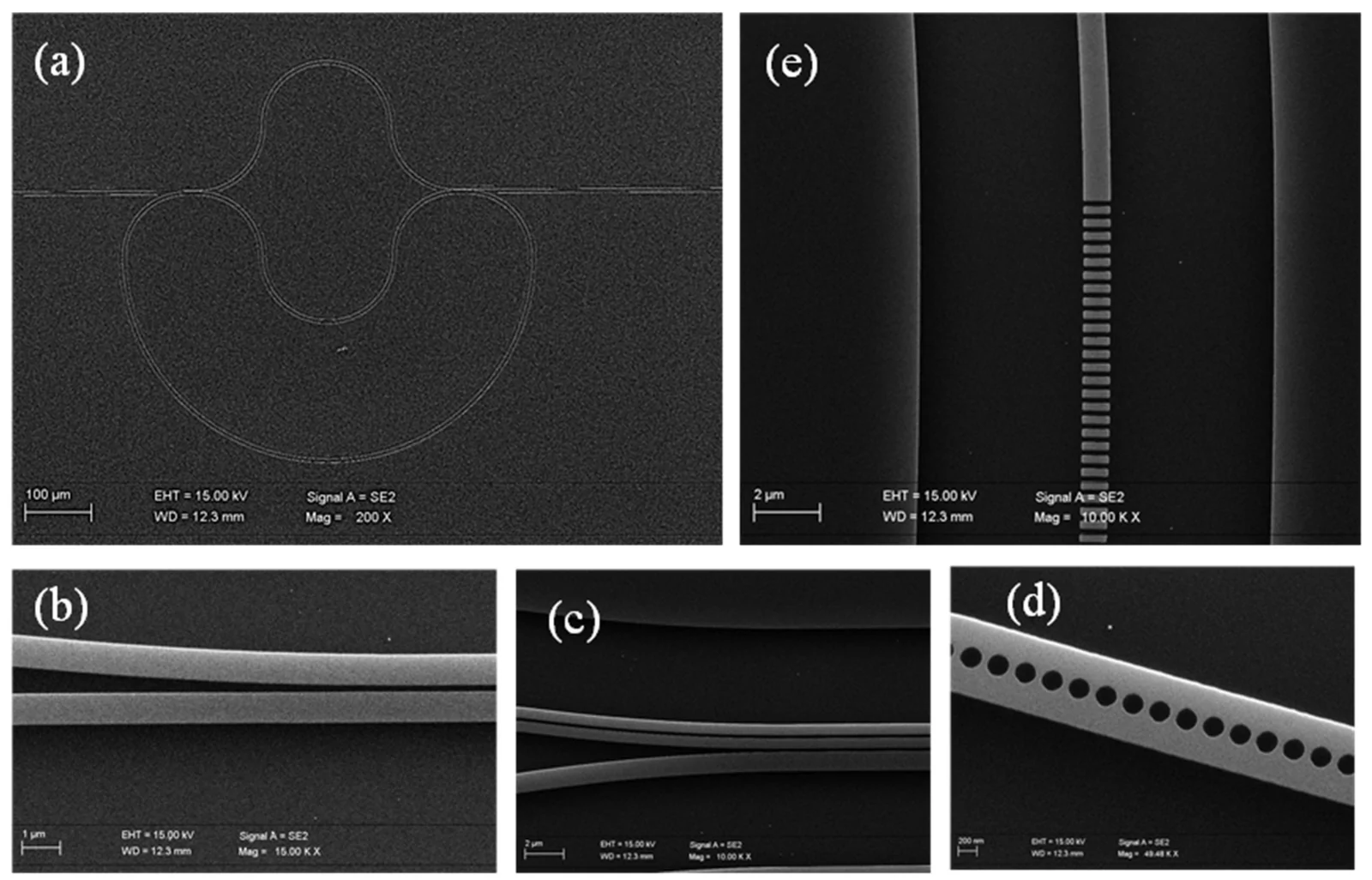
Scanning electron microscopy images of the fabricated SiN resonant-cavity sensor: (**a**) complete device; (**b**) ridge waveguide; (**c**) slot waveguide side-coupled to the bus waveguide; (**d**) photonic-crystal waveguide; and (**e**) subwavelength-grating waveguide [108].

**Figure 7:**
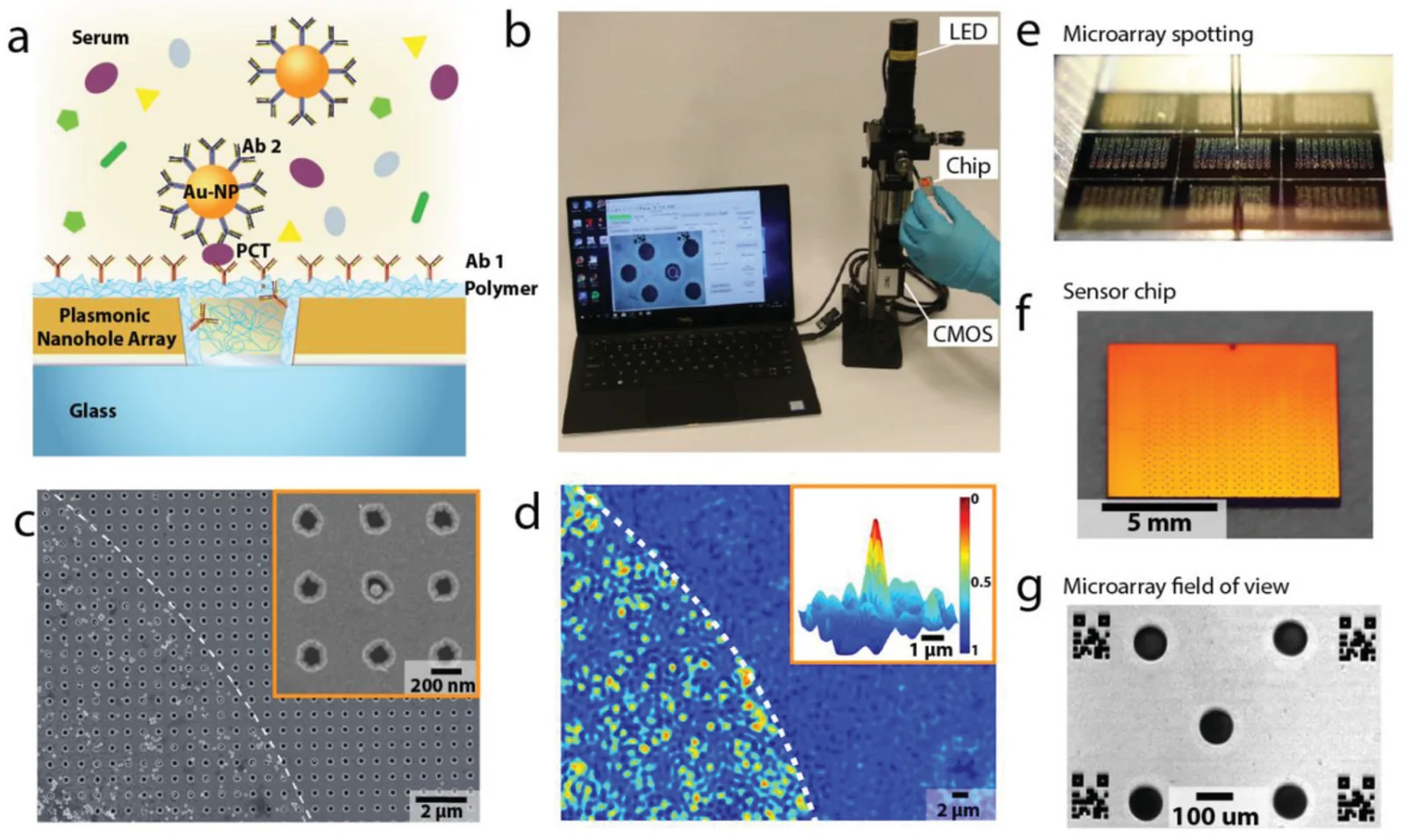
Portable digital nanoparticle-enhanced plasmonic imaging platform for rapid detection of inflammatory biomarkers in human serum: (**a**) assay principle for procalcitonin and C-reactive protein; (**b**) compact LED–CMOS optical reader; (**c**) gold nanoparticles bound to the nanohole array; (**d**) corresponding digital transmission-contrast image; (**e**) automated antibody microarray spotting; (**f**) wafer-scale-fabricated plasmonic sensor chip; and (**g**) antibody-patterned sensing field with QR-based identification [70].

**Figure 8:**
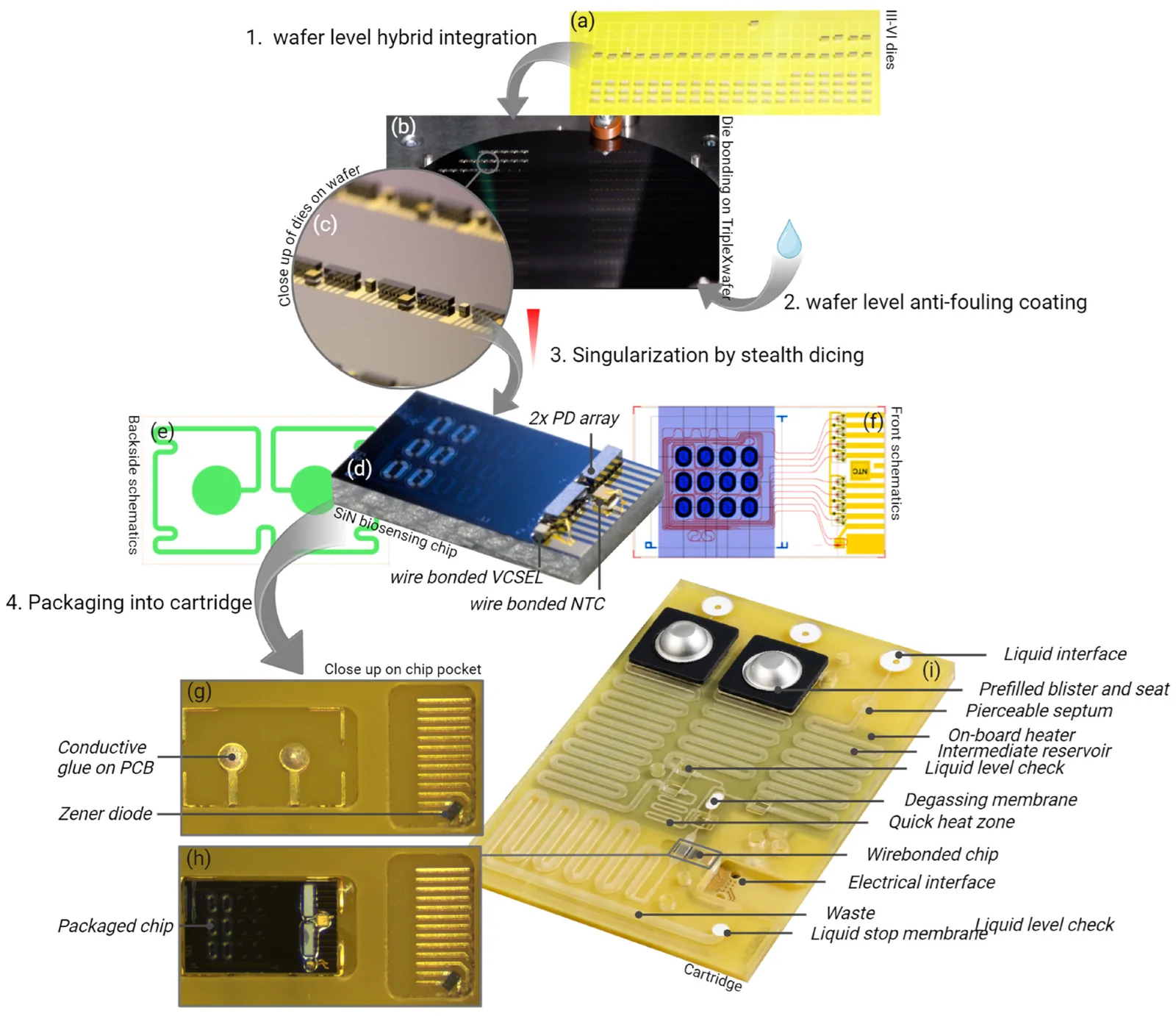
System-level integration of a SiN photonic biosensing platform for pathogen monitoring in aquaculture. (**a**,**b**) Wafer-level transfer and flip-chip bonding of GaAs photodetectors; (**c**) multiple photonic dies populated with VCSELs, photodetector arrays, and temperature sensors; (**d**) completely assembled and wire-bonded hybrid photonic chip; (**e**) backside resistive heater; (**f**) front-side waveguide architecture containing asymmetric Mach–Zehnder interferometric biosensors and auxiliary channels; (**g**) conductive-adhesive deposition within the printed-circuit-board cavity; (**h**) placement and encapsulation of the hybrid chip; and (**i**) assembled microfluidic cartridge [151].

**Figure 9:**
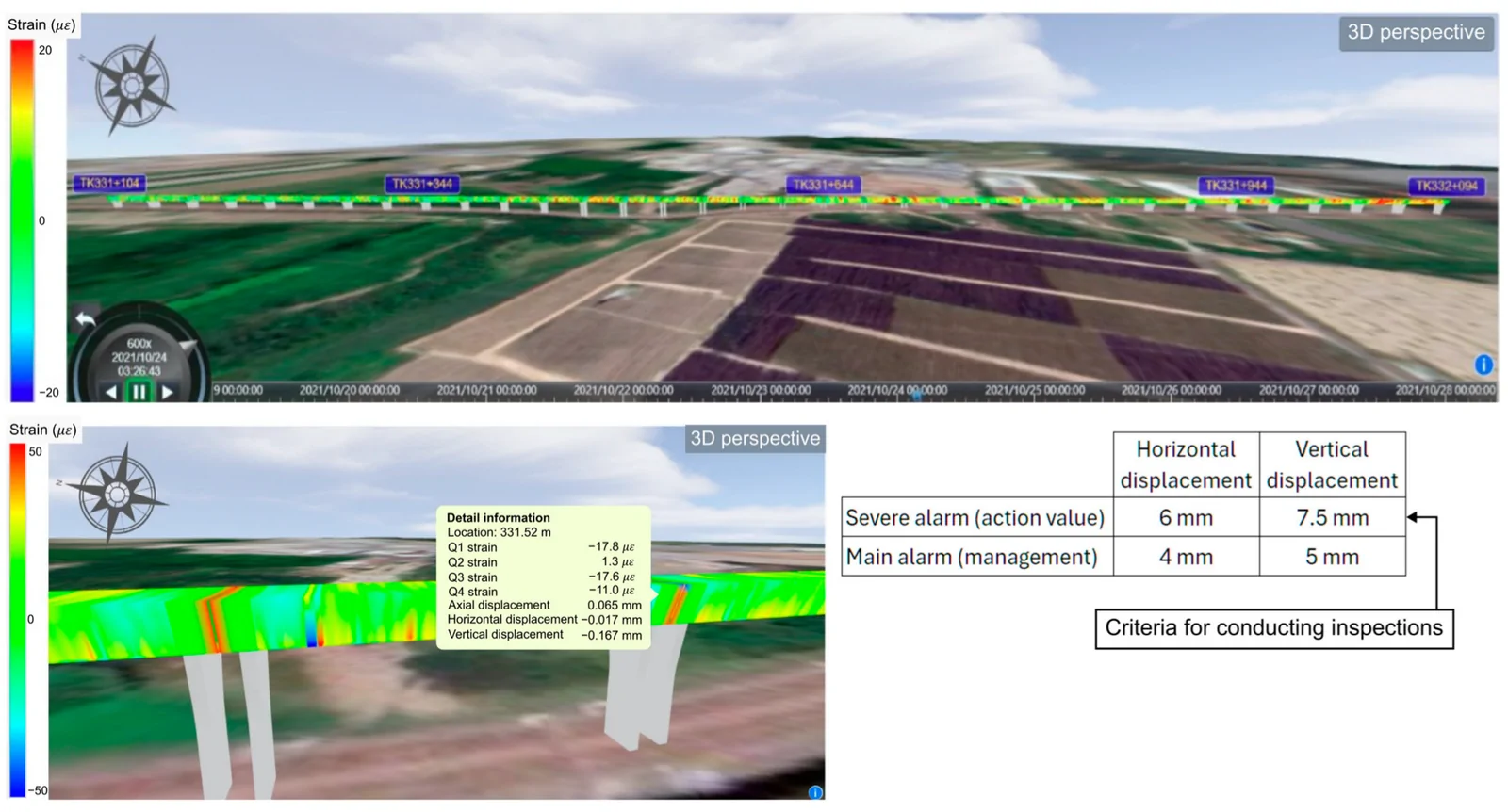
Distributed optical-fiber monitoring of an operational high-speed railway bridge. Spatially resolved strain measurements are converted into axial, horizontal, and vertical displacement profiles and integrated into a SCADA system with predefined warning and inspection thresholds [159].

**Figure 10:**
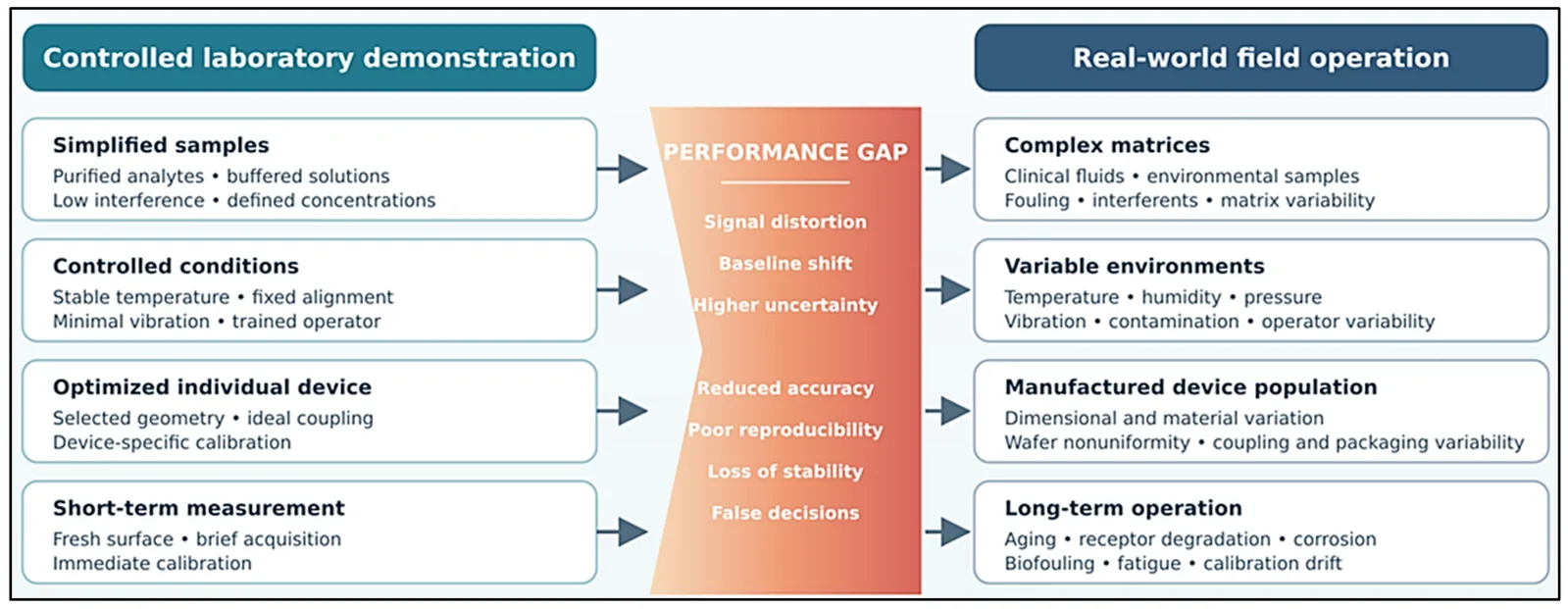
Sources of performance degradation during the translation of photonic sensors from controlled laboratory demonstrations to real-world operation.

**Figure 11:**
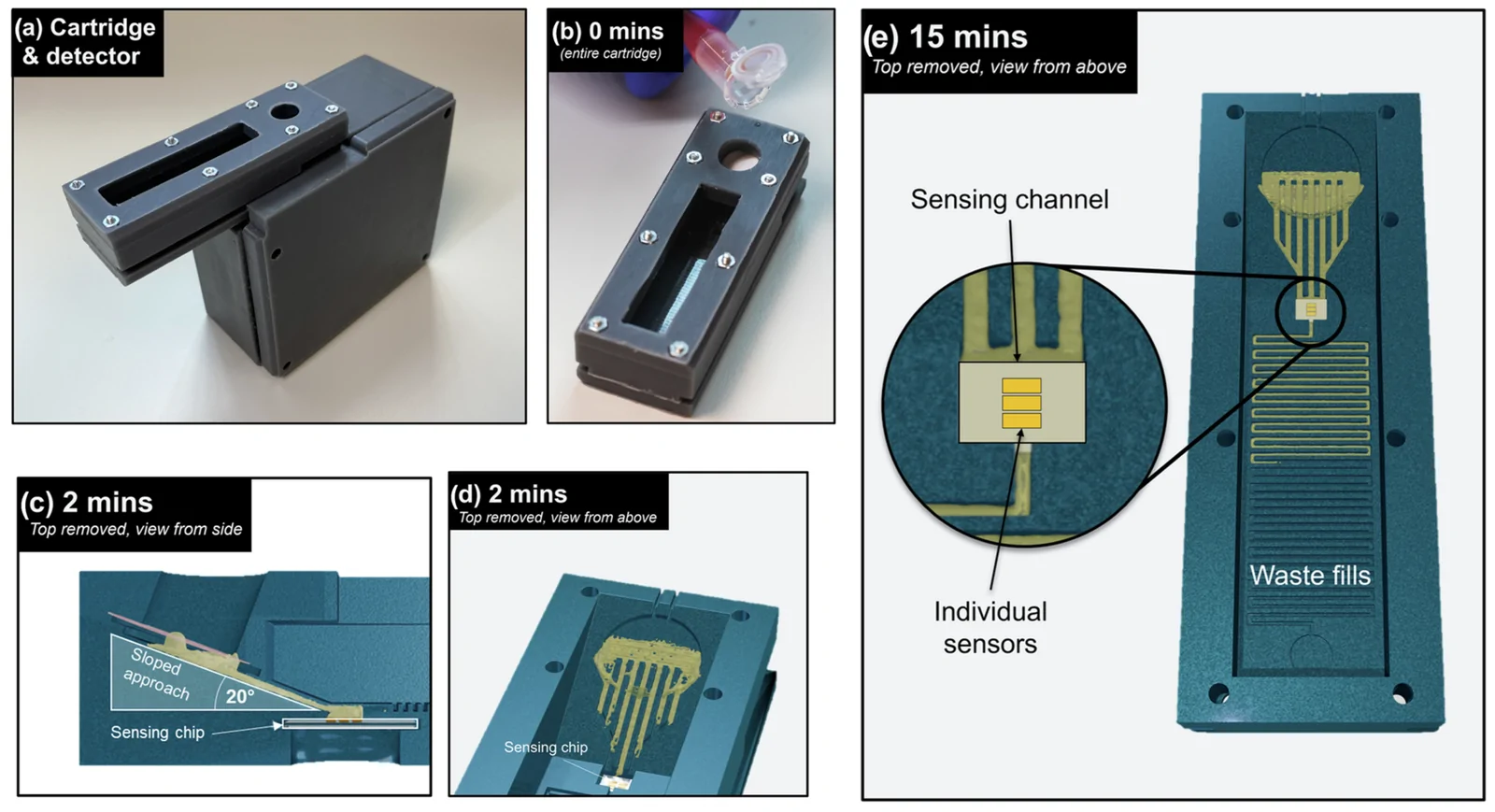
(**a**–**e**) System-level integration of passive sample preparation and photonic point-of-care sensing. The disposable cartridge combines membrane-based blood separation, gravity-assisted plasma delivery, capillary-driven transport across a Si_3_N_4_ guided-mode-resonance sensing chip and downstream waste containment within a compact optical detection platform [176].

**Figure 12:**
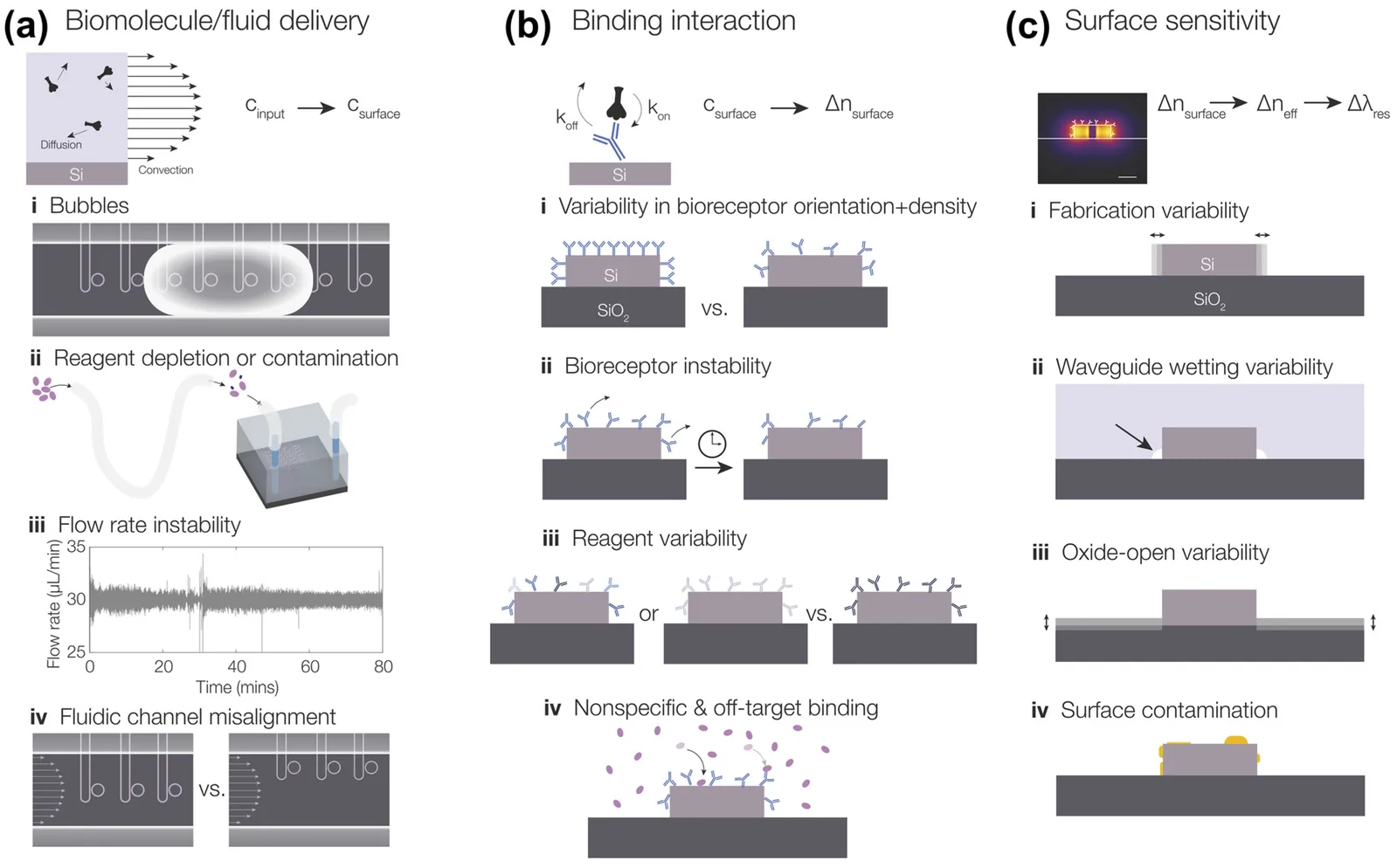
Sources of variability across the silicon-photonic biosensing measurement chain. (**a**) Biomolecule delivery to the sensor surface and associated variability arising from bubbles, reagent depletion or contamination, flow instability and fluidic-channel misalignment. (**b**) Target–receptor binding and variability related to bioreceptor orientation and density, receptor instability, reagent variability and nonspecific or off-target adsorption. (**c**) Conversion of the surface refractive-index change into a resonance shift and the influence of fabrication tolerances, waveguide wetting, oxide-opening variability and surface contamination [28].

**Figure 13:**
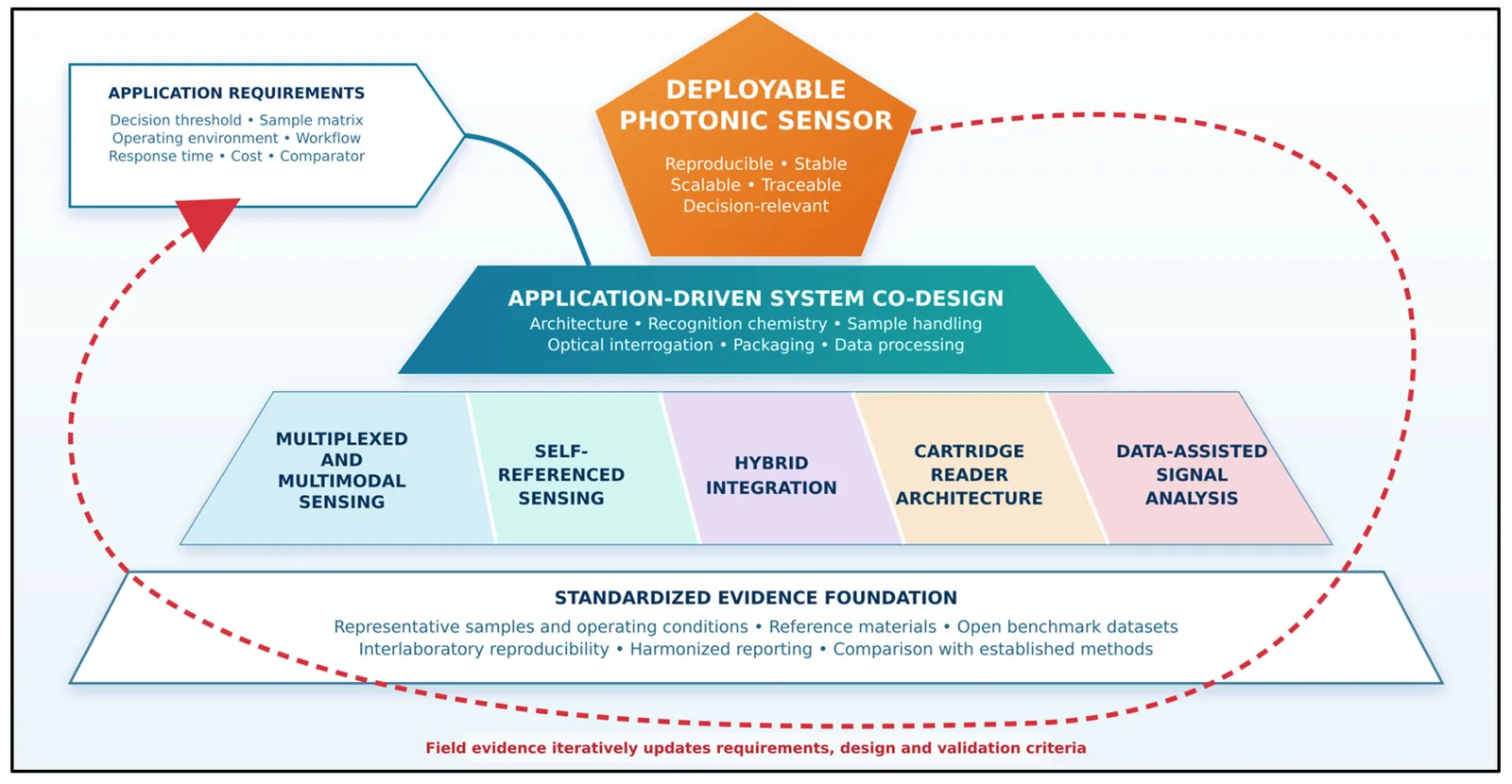
Application-driven roadmap for next-generation deployable photonic sensors, linking use-case definition, complete-system engineering, and standardized validation through an evidence-driven feedback process.

**Table 3:** Workflow-specific economic comparison of photonic biosensors, ELISA, and PCR-based assays. Entries identify cost drivers and trade-offs rather than universal price rankings.

Decision Factor	Photonic Biosensors with Disposable Cartridges and Reusable Readers	Plate-Based ELISA	Laboratory PCR or RT-qPCR
Initial setup costs	Dedicated reader and any required fluidic or sample-preparation equipment; costs depend strongly on whether interrogation uses compact illumination or precision laser instrumentation.	Plate reader and basic liquid-handling equipment; automated washing adds investment. Existing equipment can reduce incremental acquisition costs.	Thermal cycler, detection optics for real-time measurements, and sample-preparation equipment; automated extraction adds investment.
Single-use consumable costs	Functionalized chips, cartridges, recognition reagents, and controls. Multiplexing may reduce cost per target, but packaging and expired cartridges can offset savings.	Plates, antibodies, substrates, standards, and tips. Cost per reportable sample depends on plate occupancy, controls, and replicate requirements.	Extraction materials, amplification reagents, primers, probes or dyes, reaction vessels, and controls. Costs depend on reaction volume and multiplexing.
Time to result	Potential for on-demand analysis, but sample preparation, binding, washing, and interpretation must be included.	Incubation and washing determine assay duration; batch accumulation can delay reporting. Rapid ELISA formats must also be considered.	Extraction, reaction preparation, amplification, and interpretation determine turnaround; rapid integrated systems can shorten the workflow.
Long-term maintenance costs	Optical and thermal verification, software support, and servicing of reusable fluidics. Disposable fluid paths reduce cleaning requirements but increase consumable dependence.	Reader verification, pipette calibration, and washer servicing; maintenance expenses can be shared across assays.	Thermal and optical verification, extraction-system servicing, pipette calibration, and contamination control.
Conditions favoring adoption	Demonstrated savings in handling, sample volume, or time to an actionable result sufficient to justify reader and cartridge costs.	Efficient batch testing, established assays, and access to existing laboratory infrastructure.	Applications requiring validated nucleic-acid detection, amplification, and molecular specificity.

## Data Availability

No new data were created or analyzed in this study. Data sharing is not applicable to this article.
